# Cell Segmentation as Strategic Decision Making

**DOI:** 10.34133/research.1304

**Published:** 2026-06-01

**Authors:** Yunshan Zhong, Xianwen Ren

**Affiliations:** ^1^ Changping Laboratory, Beijing, China.; ^2^ Human Organ Physiopathology Emulation System, State Key Laboratory of Organ Regeneration and Reconstruction, Institute of Zoology, Chinese Academy of Sciences, Beijing 100101, China.; ^3^ Beijing Institute for Stem Cell and Regenerative Medicine, Beijing 100101, China.

## Abstract

Cell segmentation in imaging-based spatial transcriptomics (ST) relies on stained image segmentation or transcript clustering, but current methods are limited by dependence on manual annotations, sensitivity to staining quality and cell density, and lack of alignment with true transcriptomic profiles. We introduce RedeFISH, a reinforcement learning-based framework that formulates cell segmentation as a sequential strategic decision-making process. Through iterative optimization of the transcript assignment policy, RedeFISH aligns segmented cell expression profiles with single-cell expressions to achieve optimal segmentation. This approach enables staining-free, transcriptome-informed delineation of individual cells, thereby overcoming key limitations of existing methods. Benchmarking across multiple ST platforms shows that RedeFISH improves cosine similarity of segmented cell expression profiles by 9.7% on average and reduces root mean squared error (RMSE) by 13.9% compared with state-of-the-art methods. It also increases agreement between segmented and ground-truth cell regions by 19%, demonstrating improved accuracy and robustness. RedeFISH further imputes whole-transcriptome profiles from sparse ST measurements via spatially guided transfer from scRNA-seq data. The whole-transcriptome coverage enables unbiased and in situ prioritization of the critical regulators of spatial niches, e.g., mouse intestinal stem cell niches, and unlocks the possibility to delineate, with only one sample, the panoramic molecular and cellular changes along the whole developmental process from initiation to invasion for human cancer.

## Introduction

The rapid development of spatial transcriptomics (ST) technologies has provided unprecedented resolution to investigate spatial gene expression. In addition to ST platforms, e.g., 10x Genomics Visium, which measure gene expression at spatial resolution of multiple micrometers [1,2], single-molecule ST platforms achieve subcellular resolution by localizing mRNAs in situ [3–5]. Such higher resolution and limited number of detected genes raise new analytical questions, with cell segmentation and gene imputation typically as the foremost post-experiment steps pivotal to systematic investigation.

In recent years, numerous cell segmentation methods have been developed. The majority of existing approaches rely on convolutional neural networks (CNNs) for feature extraction within supervised learning frameworks, which necessitate large-scale, manually annotated histological images for model training [6,7]. Leveraging transfer learning, recent foundation models pretrained on histologically stained tissues have demonstrated improved feature representation capabilities [8–10]. However, these models still require fine-tuning with annotated datasets to enable segmentation. In practice, variations in cell density across tissue regions, inconsistent staining quality, and the lack of transcriptomic information further undermine the robustness and generalizability of these methods, and often lead to inaccurate delineation of cell boundaries. Some unsupervised approaches attempt to segment cells by clustering transcripts based on their spatial locations [11–14]. Nevertheless, these methods often overlook whether the resulting expression profiles are consistent with known single-cell transcriptomes, which may lead to the erroneous assignment of transcripts to unrelated cells. Such misassignments will further compromise downstream analyses, including differential expression analysis and ligand–receptor interaction inference [15]. Collectively, these limitations underscore the fundamental challenges faced by supervised, unsupervised, and transfer learning-based approaches in achieving robust and biologically meaningful cell segmentation.

Inspired by AlphaGo Zero [16], which demonstrated the capabilities of reinforcement learning (RL) in solving complex, high-dimensional tasks without human supervision in the field of Go, RL also offers a fundamentally different paradigm for cell segmentation by formulating it as a sequential strategic decision-making problem. To our knowledge, no prior studies have reported any attempt to apply RL to cell segmentation. In contrast to existing approaches, in our proposed RL framework, the assignment of transcripts to individual cells is modeled as a series of actions based on spatial coordinates and gene expression profiles. Through iterative policy optimization, the RL agent learns to generate segmented cells whose transcriptomic signatures closely correspond to established single-cell references, thereby achieving biologically consistent and optimal segmentation. This also highlights a fundamental difference between RL and existing methods, where RL-based segmentation relies on transcript signals and spatial information with a reward–penalty mechanism, rather than primarily depending on staining.

In this study, we developed RedeFISH, an RL-based framework that performs strategic cell segmentation and gene imputation for imaging-based ST data with the assistance of single-cell RNA sequencing (scRNA-seq) data. Within this framework, an RL agent is defined to iteratively refine its policy for transcript assignment and cell boundary delineation, aiming to optimize transcriptomic profiles that closely match reference single-cell expression signatures. Analogous to AlphaGo Zero, which surpassed human performance in Go by learning from self-play without relying on human game records, RedeFISH employs RL to autonomously derive an optimal cell segmentation policy directly from ST data, without relying on stained images for supervision. This not only obviates the need for annotated image pretraining but also enhances robustness to staining variability and spatial tissue heterogeneity. Extensive benchmarking across diverse spatial platforms and tissue types demonstrates that RedeFISH consistently outperforms state-of-the-art (SOTA) methods in terms of segmented cell expression profiles, cell coverage regions, and cellular morphology, highlighting its superior accuracy and robustness. RedeFISH further enhances subcellular ST by extending coverage from hundreds of genes to the entire transcriptome, facilitating biological investigations through spatially transferable mapping of scRNA-seq data. Application of RedeFISH to a mouse ileum dataset elucidated Wnt/β-catenin signaling dynamics within intestinal stem cell niches, while analysis of a human breast cancer dataset revealed the transition from ductal carcinoma in situ (DCIS) to invasive carcinoma. By leveraging RL, RedeFISH substantially advances cell segmentation and gene imputation, thereby enabling more accurate and comprehensive ST analyses that deepen our understanding of complex tissue biology.

## Results

### The design of RedeFISH

RedeFISH is the first method to introduce RL to cell segmentation for ST data. RL offers a powerful framework for cell segmentation by formulating it as a sequential strategic decision-making process. Within this framework, the state represents the current configuration of transcript assignments, while actions correspond to allocating individual transcripts to cells based on spatial coordinates and gene expression features. The agent learns an optimal policy through iterative interactions with the environment, guided by a reward function that measures the similarity between the transcriptomic profiles of segmented cells and known single-cell references.

**Fig. 1.**
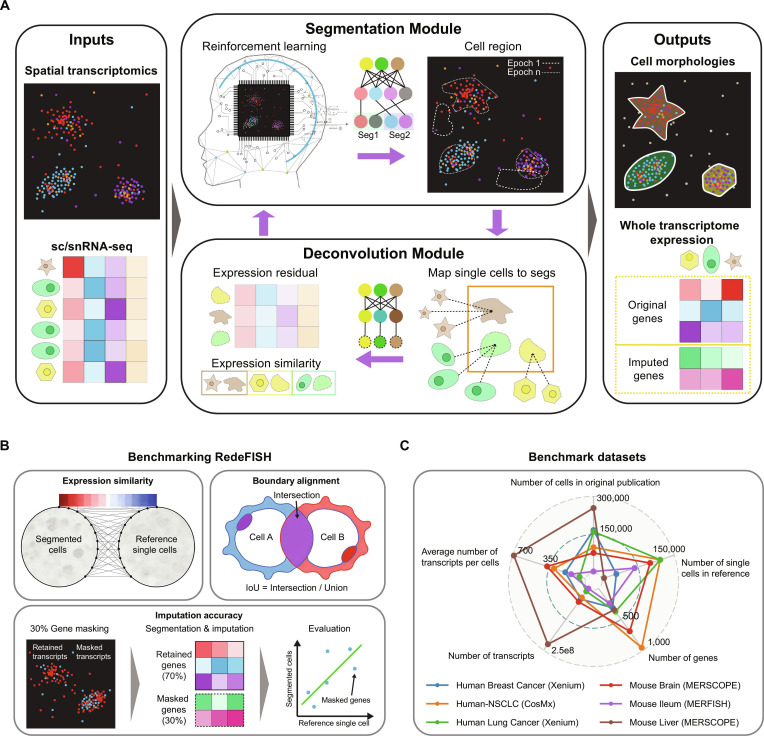
Overview of the RedeFISH framework and the benchmarking strategy for performance evaluation. (A) The overview workflow of the RedeFISH algorithm. RedeFISH integrates sc/snRNA-seq with spatial transcriptomics data within an RL framework for simultaneous cell segmentation and gene imputation. The framework employs 2 core components: (1) a Segmentation Module that assigns transcripts to cells using spatial and expression patterns, and (2) A Deconvolution Module that assesses transcriptomic similarity to reference profiles as part of the reward function. Through iterative optimization, the system converges to biologically plausible cell segmentation. The final output of the model consists of segmented cell regions and their corresponding whole-transcriptome expression profiles. (B) Benchmarking of RedeFISH, including evaluation of the similarity between segmented cells and reference single cells, assessment of cell boundary consistency, and validation of imputation accuracy through random gene masking. Some graphical elements were partially generated using Doubao AI and further refined by the authors. (C) Summary of the 6 datasets used for benchmarking RedeFISH, including the total number of transcripts, number of cells, average transcripts per cell, number of genes, and the number of reference single cells in each dataset.

The overall architecture of RedeFISH is illustrated in Fig. 1A. RedeFISH takes ST and single-cell or single-nucleus RNA sequencing (sc/snRNA-seq) data as input, and outputs segmented cells along with their whole-transcriptome expression profiles. In RedeFISH, the Segmentation Module formulates transcript-to-cell assignment as an RL problem that jointly leverages spatial coordinates and gene expression patterns. In detail, an action space is defined as the set of all possible cell assignment decisions for each transcript. At each step, an action corresponds to assigning a specific transcript to a candidate cell (or a “background”/null class) based on the transcript’s spatial coordinates and its gene expression profile. The agent employs a multi-modal fusion architecture that integrates transcript spatial coordinates, candidate cell embeddings, and gene features through a series of linear transformations and residual connections, followed by a categorical policy head that outputs assignment probabilities over candidate cells. The reward function is defined based on the multiplicative integration of transcript spatial proximity, expression similarity, and local transcript density. This formulation ensures that transcripts assigned to segmented cells simultaneously satisfy spatial adjacency, high-density regions, and expression profiles consistent with reference single-cell transcriptomes. The module is trained using a policy gradient-based framework to learn an optimal assignment policy. The Deconvolution Module adopts a deconvolution-based strategy to estimate the expected expression profile of each segmented cell using reference single-cell transcriptomes and an estimated cell abundance matrix. As ST and sc/snRNA-seq data may exhibit batch effects, we derive cell type-level expression profiles from single-cell transcriptomes and use them as references for deconvolution. A neural network is employed to learn cell-specific weights, enabling adaptive adjustment of the reference expression profiles and thereby mitigating potential batch discrepancies between the cell type signatures and the ST data. The predicted profiles are then compared with the expression profiles obtained from the Segmentation Module, and the resulting discrepancy is incorporated into the reward function. This design encourages transcript assignments that drive segmented cell expression profiles toward greater consistency with the deconvolution-based estimates. Through iterative interaction between these modules, RedeFISH progressively refines transcript assignments, enabling the framework to converge toward an optimal and biologically meaningful cell segmentation. After an optimal cell segmentation result is obtained, the whole-transcriptome gene expression profiles of scRNA-seq data are transferred to the corresponding single cells segmented from ST data, raising the ST measurement from hundreds of genes to whole transcriptomes and enabling genome-wide association analyses.

### RedeFISH demonstrates robust superior performance on various ST platforms

We evaluated the performance of RedeFISH from 3 perspectives, namely, the similarity of segmented cell expression profiles, the consistency of inferred cell boundaries, and the accuracy of imputed gene expression (Fig. 1B). The evaluation was conducted on 6 ST datasets spanning 4 representative platforms, including MERFISH, Xenium, CosMX, and Stereo-seq, across different species (human and mouse) and multiple organs, including ileum, breast, brain, liver, and lung (Fig. 1C). Compared with cell segmentation results generated by Baysor [11] and Proseg [13], 2 SOTA algorithms, as well as Cellpose-SAM [17], Stardist [7], and manual segmentations reported in the original studies using staining data, RedeFISH consistently demonstrated superior and robust performance in the quality of the identified cells. These results validate the accuracy of the cell segmentation and demonstrate the feasibility of generating cell-resolved, whole-transcriptome spatial data through automated cell segmentation and gene imputation of imaging-based ST data, guided by scRNA-seq references.

**Fig. 2.**
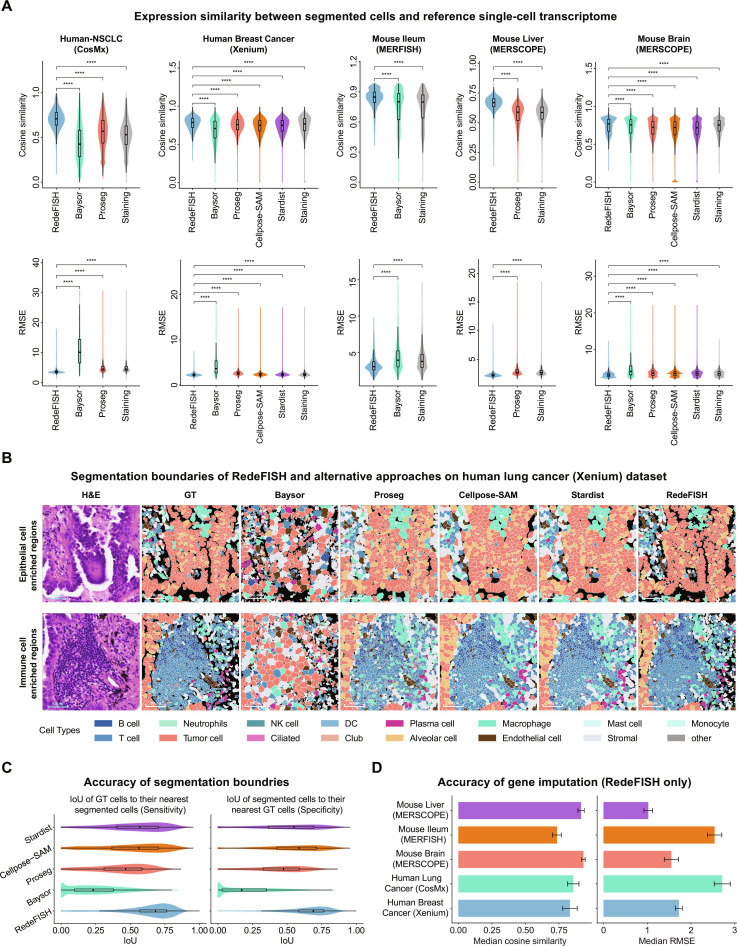
Benchmarking for RedeFISH and alternative approaches. (A) Cosine similarity and RMSE between expression profiles of segmented cells and reference single-cell transcriptomes for RedeFISH and alternative approaches. (B) Cell segmentation boundaries of RedeFISH, GT, and alternative approaches on the Human Lung Cancer (Xenium) dataset. Cell types were determined via label transfer. (C) Quantitative evaluation of cell boundary accuracy for RedeFISH and alternative approaches by comparing the Intersection over Union (IoU) between segmented cells and GT cell coverage. (D) Evaluation of RedeFISH imputation accuracy across 5 datasets using Cosine similarity and RMSE. The boxplot’s and violin plot’s center line indicates the median, while the box spans Q1 to Q3. Whiskers extend to 1.5 × IQR (Q3 − Q1), with points beyond considered outliers. Min/max bounds are Q1 − 1.5 × IQR and Q3 + 1.5 × IQR. The significance level marker denotes the level of significance under the null hypothesis. “*”, “**”, “***”, and “****” denote significance levels of less than 0.05, 0.01, 0.001, and 0.0001, respectively. Error bars in the bar plots represent ±1.96 standard deviations.

First, RedeFISH achieves precise alignment between ST segments and single-cell transcriptomes, as evidenced by superior gene expression concordance. Across all 5 evaluated datasets, RedeFISH consistently outperformed Baysor, Proseg, Cellpose-SAM, Stardist, and staining-based methods, achieving an average cosine similarity 9.7% higher than the second-best method and an average root mean squared error (RMSE) 13.9% lower (Fig. 2A). These results highlight its ability to generate biologically reliable single-cell expression profiles, thereby strengthening the interpretability and accuracy of downstream transcriptomic analyses.

Second, we evaluated boundary accuracy using a human lung cancer Xenium dataset. In the original study, nuclear, interior, and membrane staining were performed, and accurate cell boundaries were obtained through multimodal cell segmentation. The resulting boundaries were treated as ground truth (GT), and we compared the consistency of cell boundaries inferred by RedeFISH and alternative approaches with the GT. Similar to Proseg, which uses Xenium transcript assignments as a prior, RedeFISH incorporates Xenium nuclei regions as prior information. In epithelial cell-enriched regions, RedeFISH achieved high concordance with GT boundaries, whereas Cellpose-SAM and Stardist tended to overexpand cell areas, Proseg produced mosaic-shaped boundaries, and Baysor generated cells with markedly divergent sizes and distributions. In immune cell-enriched regions, RedeFISH accurately delineated immune cells and vascular structures surrounded by endothelial cells, while Cellpose-SAM and Stardist overestimated endothelial cell boundaries, and Proseg and Baysor exhibited substantial deviations from the GT (Fig. 2B). Quantitative evaluation cell regions using intersection over union (IoU) revealed that RedeFISH consistently outperformed all alternative approaches, achieving a 19.5% higher sensitivity and an 18.9% higher specificity compared to the second-best method, demonstrating superior boundary accuracy across both metrics (Fig. 2C). We further assessed the consistency of morphological features between segmented cells and GT cells. In RedeFISH, the density distributions of cell area and girth closely resembled those of the GT, whereas the distribution of roundness exhibited partial agreement (Fig. S1A). To quantify these differences, we computed the IoU between the areas under the density curves for each method and the corresponding GT distributions across morphological features. RedeFISH outperformed the second-best method by 21.8% and 20.4% in area and girth, respectively, while achieving roundness performance comparable to that of the best-performing method (Fig. S1B). We next estimated the distribution of noise transcripts, which predominantly originated from intercellular spaces or regions outside the tissue (Fig. S1C and D). Specifically, in regions enriched with epithelial or immune cells, cellular and tissue architectures are well-defined, and transcript density is high, facilitating accurate cell segmentation. In contrast, stromal-enriched regions, such as the lung pleura, exhibit thin structures, low transcript density, and poorly demarcated boundaries with the thoracic cavity, which pose significant challenges to all segmentation approaches and increase noise transcript prevalence. The impact of noise sampling on segmentation performance in RedeFISH was also assessed. Although removing noise sampling led to an overall increase in the transcript assignment rate, it reduced the consistency of both cell regions and morphological features (Fig. S1E), indicating that noise sampling has a beneficial effect on segmentation performance.

Third, we evaluated the accuracy of RedeFISH for gene imputation. For each dataset, 70% of genes were randomly selected for cell segmentation, and the remaining 30% were imputed and compared with reference single-cell expression profiles. This procedure was repeated 3 times. RedeFISH achieved cosine similarity values exceeding 0.8 in most datasets, together with low RMSE, demonstrating high imputation accuracy (Fig. 2D).

Fourth, RedeFISH produced cell counts across multiple datasets that closely matched those obtained from staining-based segmentation, whereas Baysor occasionally generated more than twice as many cells in certain datasets (Fig. S2A). In addition, image-based segmentation methods exhibit substantial variability in transcript assignment rates across ST platforms, suggesting that some methods may perform suboptimally on certain datasets and yield unreliable expression profiles for segmented cells. In contrast, RedeFISH, Proseg, Cellpose-SAM, and Stardist maintain consistently high transcript assignment rates across platforms, indicating more stable performance (Fig. S2B). Meanwhile, for difficult-to-segment transcripts (e.g., stromal/extracellular matrix-related genes), RedeFISH exhibits relatively higher assignment rates (Fig. S2C). Regarding computational efficiency, although RL methods typically require substantial computational resources, optimization of the RedeFISH pipeline enables processing of ST datasets containing tens of millions of transcripts within 2 to 3 hours on a single graphics processing unit (GPU) (Fig. S2D). Furthermore, we extended RedeFISH to support sequencing-based ST platforms and evaluated its performance on a mouse brain dataset generated using Stereo-seq, demonstrating its strong generalizability to sequencing-based ST data (Fig. S2E).

These evaluations demonstrate that RedeFISH achieves superior accuracy and robustness across datasets in terms of cell expression profiles, cell boundary delineation, and gene imputation, producing segmented cells with both consistent cell numbers and reliable quality.

### RedeFISH enables precise characterization of tissue architectures

**Fig. 3.**
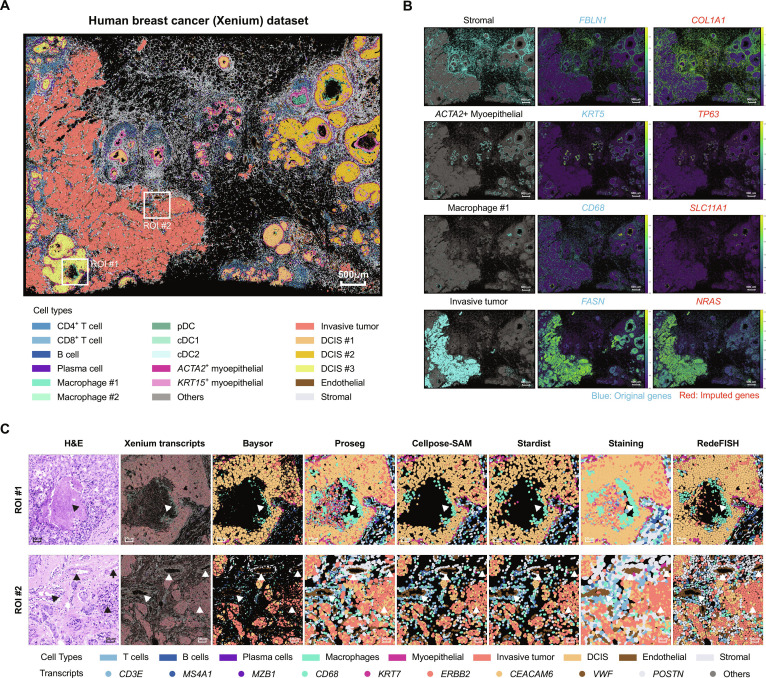
RedeFISH reveals human breast cancer architecture in a Xenium dataset. (A) Spatial distribution of all cell types. (B) Spatial distribution of stromal cells, *ACTA2*^+^ myoepithelial cells, macrophage #1, and invasive tumor cells with original and imputed marker genes. (C) Comparison of H&E-stained image, Xenium transcript distributions, and cell segmentation results from RedeFISH and alternative approaches across 2 ROIs. pDC, plasmacytoid dendritic cell; cDC1, conventional dendritic cell type 1; cDC2, conventional dendritic cell type 2; DCIS, ductal carcinoma in situ.

We illustrate how RedeFISH uncovers spatial tissue structures by performing cell segmentation of ST data through decision-making strategies guided by scRNA-seq. We began by evaluating the performance of RedeFISH on a human breast cancer dataset generated by the 10x Genomics Xenium platform [5], which comprises a HER2^+^/ER^+^/PR^−^ tissue block and contains multiple regions of DCIS and invasive tumors. By annotating the segmentation results, RedeFISH resolved the spatial organization of diverse cell types, including B cells, T cells, invasive tumor cells, and DCIS cells (Fig. 3A and Fig. S3). Notably, the spatial distributions of major cell populations exhibited strong concordance with Xenium-detected marker gene expression, as well as with additional marker genes imputed by RedeFISH that were not assayed by Xenium (Fig. 3B). This concordance underscores the accuracy of RedeFISH in both cell segmentation and transcriptome-wide imputation while extending beyond experimentally measured panels to provide a more comprehensive view of tissue architecture.

A comparison of label transfer results from RedeFISH, Baysor, Proseg, Cellpose-SAM, Stardist, and staining-based approaches in regions of interest (ROI) #1 showed that all methods successfully identified ductal structures and DCIS cells. However, due to the intensely stained central ductal region in the hematoxylin and eosin (H&E) image, Proseg and staining-based methods erroneously inferred numerous cells in this area, whereas ST data revealed minimal transcript accumulation (Fig. 3C). Baysor and RedeFISH, which do not rely on staining images for segmentation, effectively avoided this artifact. Nonetheless, Baysor generated overly fragmented DCIS cell segments, leading to incomplete reconstruction of the ductal structure, while Cellpose-SAM and Stardist introduced numerous artificial gaps within the DCIS cell regions. In ROI #2, both RedeFISH and Proseg accurately delineated the vascular structures surrounding invasive tumor cells (Fig. 3C). By contrast, staining-based methods exhibited inaccurate cell boundary delineation, preventing clear visualization of the vasculature, and Baysor, Cellpose-SAM, and Stardist produced incomplete vascular structures, with Baysor additionally generating overly fragmented adjacent invasive tumor cells.

**Fig. 4.**
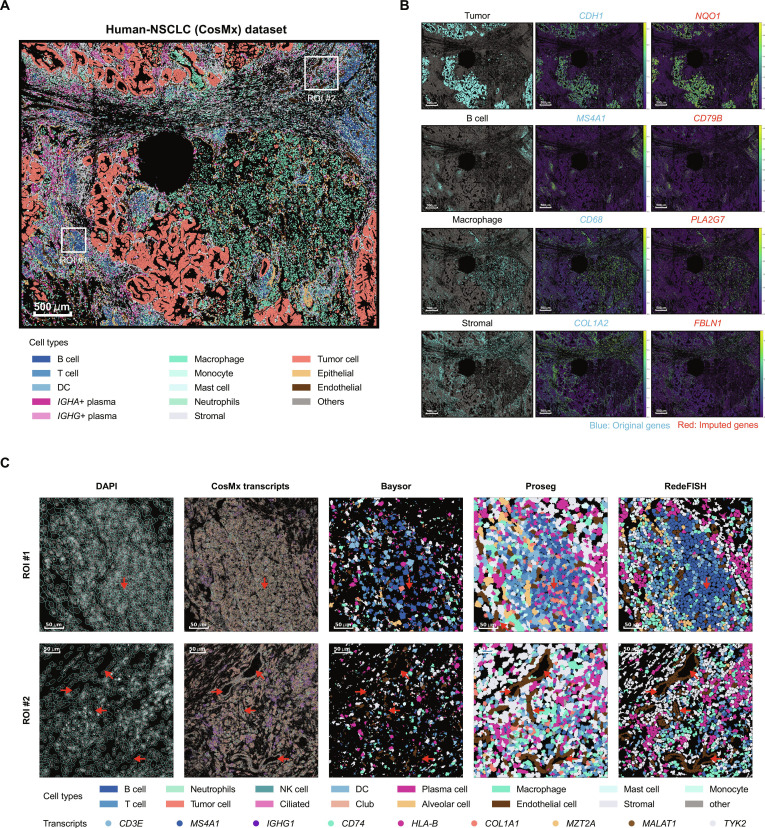
RedeFISH reveals human-NSCLC architecture in a CosMx dataset. (A) Spatial distribution of all cell types. (B) Spatial distribution of tumor cells, stromal cells, macrophage, and B cells with original and imputed marker genes. (C) Comparison of DAPI-stained image, CosMx transcript distributions, and cell segmentation results from RedeFISH and alternative approaches across 2 ROIs. DC, dendritic cell.

We next evaluated RedeFISH on a human non-small cell lung cancer (NSCLC) dataset generated by the Nanostring CosMx platform [4], which captured ~150 mm^2^ of lung tissue with identifiable tumor regions and tertiary lymphatic structures. Similarly, RedeFISH accurately captured the spatial distribution of various cell types, including tumor cells, epithelial cells, endothelial cells, and immune cells (Fig. 4A and Fig. S4). The concordance between cell type distributions and marker gene expression further validates the reliability of RedeFISH’s cell segmentation and gene imputation on this dataset (Fig. 4B). For comparative analysis, in a tertiary lymphoid structure (TLS) region (ROI #1), both Proseg and RedeFISH identified densely clustered immune cells (Fig. 4C). However, in the Proseg results, expression-based label transfer misclassified many TLS-resident cells as tumor cells. By contrast, RedeFISH employs transcriptome-driven cell segmentation, yielding more accurate single-cell expression profiles and preserving the TLS as a region predominantly composed of immune cells. In ROI #2, both RedeFISH and Proseg effectively reconstructed the vascular structures, whereas Baysor again exhibited issues with fragmented cell segmentation (Fig. 4C).

On a mouse ileum ST dataset generated by the MERFISH platform, which profiled the spatial distribution of 241 genes, RedeFISH identified clear histological structures consistent with intestinal anatomy, whereas the Baysor algorithm struggled to distinguish the distribution regions of stem cells and transit-amplifying cells (Fig. S5). On a mouse brain MERSCOPE dataset, RedeFISH effectively resolved the spatial distribution of diverse brain cell types. In comparison, Baysor and Proseg exhibited notable limitations, with Baysor generating incomplete tissue structures and Proseg showing reduced accuracy near stitched image boundaries. Cellpose-SAM erroneously detected numerous cells outside the brain tissue, whereas Stardist failed to capture cells in the hippocampal region (Fig. S6). On a mouse liver dataset generated by the MERSCOPE platform, of which a total of 347 genes were profiled, RedeFISH also yielded excellent performance similar to that of staining-based approaches, with boundaries between the pericentral and periportal regions of hepatocytes clearer in RedeFISH segmentation (Fig. S7). Similarly, RedeFISH can also be applied to sequencing-based ST data generated by the Stereo-seq platform. On a mouse brain dataset generated by Stereo-seq, with cortex and subcortical regions of various neuronal and non-neuronal cells included, RedeFISH successfully revealed the hierarchy pattern of different cell types, including excitatory glutamatergic neurons and several non-neuronal cells (astrocytes, oligodendrocytes, microglia, etc.) (Fig. S8).

### RedeFISH enables whole-transcriptome spatial analysis and uncovers Wnt/β-catenin signaling in intestinal stem cell niches

**Fig. 5.**
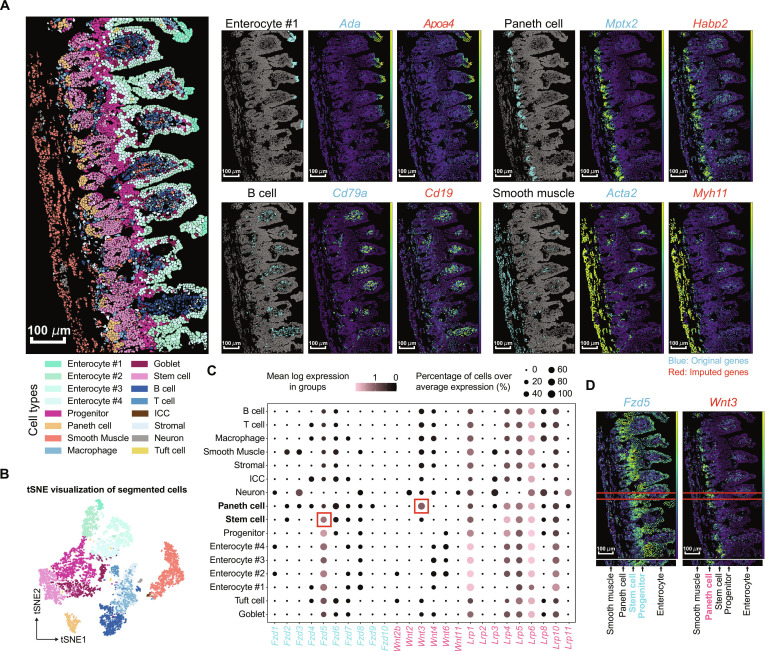
RedeFISH enables whole-transcriptome spatial analysis and reveals Wnt/β-catenin signaling dynamics in intestinal stem cell niche regulation. (A) Spatial distribution pattern of cell types and marker genes within the Mouse Ileum (MERFISH) dataset. Left: Cell type distribution through manual annotation. Right: Distribution of enterocytes #1, Paneth cells, smooth muscle cells, and B cells, along with their corresponding original and imputed marker genes. (B) tSNE plot of the cell types for the Mouse Ileum dataset. (C) Expression of *Wnt*, *Fzd*, and *Lrp* in cell types. (D) Spatial distribution of *Fzd5* and *Wnt3* as well as hierarchical pattern of cell types. ICC, interstitial cells of Cajal.

Cell segmentation of ST data with the aid of scRNA-seq by RedeFISH not only enables staining-free cell segmentation but also enables whole-transcriptome spatial expression analysis that is impossible based on ST data only. Wnt/β-catenin signaling has been shown to play important roles in intestinal stem cell maintenance, differentiation, and upon deregulation [18], but in situ investigation of Wnt/β-catenin signaling directly based on ST data is currently difficult. For example, a mouse ileum ST dataset generated by the MERFISH platform profiled the spatial distribution of 241 genes, of which 12 genes were related to Wnt/β-catenin signaling (11.3% covered, according to the Gene Ontology annotation). We applied RedeFISH to this mouse Ileum dataset and identified the clear histological structures consistent with intestinal anatomy via staining-free cell segmentation (Fig. 5A and B). The ST segments generated by RedeFISH showed consistent marker gene expression similar to scRNA-seq observation (Fig. S9A). In addition to cell markers explicitly designed by MERFISH, RedeFISH automatically upgraded the spatial data of 241 genes to the whole transcriptome and identified more genes that are cell type-specific and spatially informative (Fig. 5A). With the imputation by RedeFISH, in situ investigation of the spatial regulatory mechanisms of Wnt/β-catenin signaling in intestinal stem cell maintenance and differentiation becomes possible (Fig. S9B). Consistent with previous studies, *Wnt3* (not measured by MERFISH but imputed by RedeFISH) was highly expressed in Paneth cells (Fig. 5C and D). *Fzd5*, encoding a receptor of *Wnt* ligand and measured by MERFISH, was highly expressed in stem and progenitor cells (Fig. 5C and D). The spatial distribution of *Wnt3* (imputed by RedeFISH) and *Fzd5* (measured by MERFISH) showed the potential regulatory roles of Paneth cells in intestinal stem cell maintenance and differentiation, consistent with previous finding that Paneth cells are essential components of intestinal stem cell niches [19]. The whole-transcriptome imputation by RedeFISH enables in situ investigation of more spatial regulatory mechanisms, as exemplified by the diverse combinatory interactions between *Wnt* gene-encoding molecular components and their ligands/receptors (Fig. S9B).

### RedeFISH reveals breast cancer progression from DCIS to invasion via whole-transcriptome spatial analysis

**Fig. 6.**
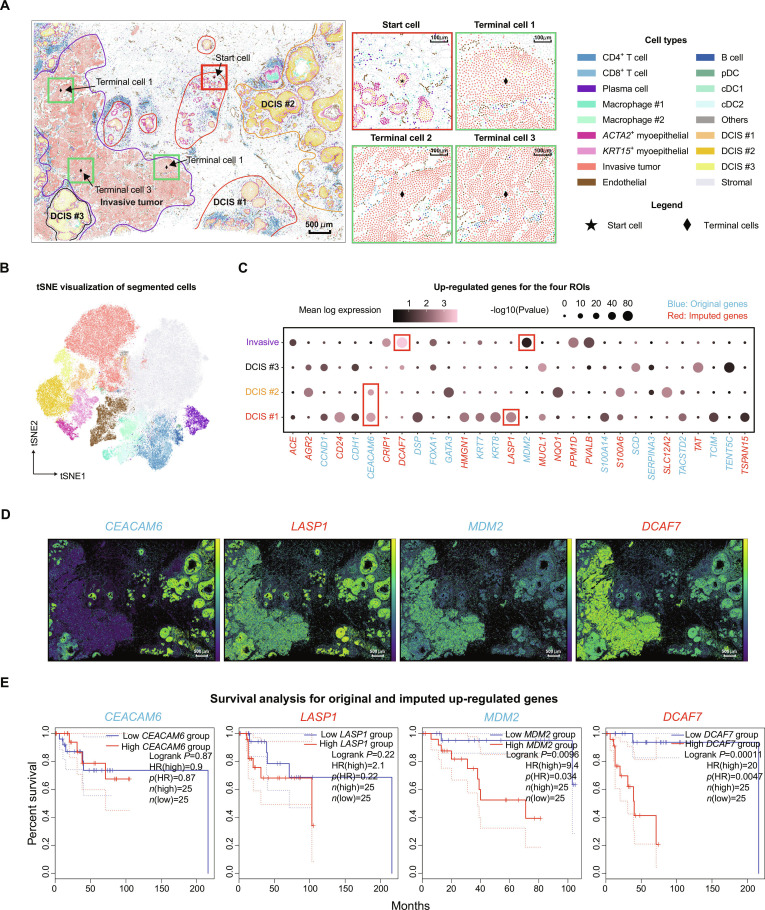
RedeFISH delineates microenvironmental and molecular dynamics during breast cancer progression from DCIS to invasion. (A) Four ROIs were defined based on invasive tumor and DCIS cell types, and appropriate start and terminal cells were selected for pseudotime analysis. (B) tSNE plot of the cell types in Human Breast Cancer dataset. (C) Up-regulated genes for DCIS and invasive tumor cells in the corresponding ROIs. (D) Spatial distribution of gene expression pattern of *CEACAM6*, *LASP1*, *MDM2*, and *DCAF7*. (E) Survival curves for *CEACAM6*, *LASP1*, *MDM2*, and *DCAF7*.

We further applied RedeFISH to the breast cancer ST dataset to investigate how tumor microenvironment changed along the transformation trajectory from ductal cells to invasive tumor cells based on the whole-transcriptome segmented cells in the Xenium ST data enhanced by our algorithm. With RedeFISH results, we first identified cell types/states for the segments of the human breast cancer Xenium dataset based on the accompanied scRNA-seq data, and identified that ductal cells in different stages of transformation were found within the same Xenium slide. We selected 4 ROIs to capture the full spectrum of ductal cell transformation, named as DCIS #1, DCIS #2, DCIS #3, and invasive tumor cells (Fig. 6A and B). Based on the whole-transcriptome expression matrix imputed by RedeFISH, we performed differential gene expression (DGE) analysis and identified specifically up-regulated genes in DCIS and invasive ROIs (Fig. 6C and D). We found that *CEACAM6* and *LASP1* were up-regulated in DCIS cells, which have been reported to promote tumor cell migration and invasion [20,21] but have no significant influence on survival rate for double-positive ERBB2^+^/ESR1^+^ (HER2^+^/ER^+^) breast cancer [22] (Fig. 6E). In contrast, *MDM2*, which plays important roles in the regulation of cellular p53 activity by a dual mechanism of degradation and transcriptional repression [23], showed association with breast cancer prognosis (Fig. 6E). Moreover, it has recently been reported that the overexpression of *DCAF7* is associated with poor prognosis in patients with pancreatic neuroendocrine tumors through degradation of MEN1 protein [24]. Consistently, we observed a significant association between up-regulated *DCAF7* and survival rate in patients with breast cancer (Fig. 6E).

In addition, we performed pseudo-time analysis based on Palantir [25] on the RedeFISH-enhanced data, and depicted a transformation trajectory from DCIS #1, DCIS #2, DCIS #3 to invasive tumor cells (Fig. 6A and Fig. S10A), consistent with the fact that DCIS is a preinvasive form of breast cancer [26]. As expected, DCIS #3 displayed a notably elevated median pseudotime (0.474) in comparison to DCIS #1 (0.198) or DCIS #2 (0.053), and aberrant duct morphology was indicated by the RedeFISH-enhanced data, suggestive of a transitioning state toward an invasive disease. In contrast, DCIS #1 and DCIS #2 were characterized by circular duct-like structures (Fig. 6A), consistent with the pseudotime analysis results (Fig. S10A). Combining pseudotime and DGE analysis, *MDM2* and *DCAF7* exhibited significant up-regulation along the transformation trajectory (Fig. S10B), consistent with the recent report of poor prognosis in various types of tumors [24,27]. These genes exhibited significant correlation with tumor development, thereby offering promising potential as biomarkers for investigating how tumor microenvironment changed along the transformation trajectory.

We finally investigated cell type composition in the tumor-adjacent regions and linked the cellular proportion changes with the corresponding pseudotime and the marker genes. Previous studies have shown that the transition from DCIS to invasive tumor cells is accompanied by cellular composition and gene expression alteration [28]. Our observations indicate that this transition involves a loss of surrounding myoepithelial cells and an increase in vascular endothelial cells, consistent with earlier report [26] (Fig. S10C). In addition, within a 50-μm spatial scale, we observed increased recruitment of macrophages to the invasive tumor site (Fig. S10D), which may modulate the tumor microenvironment to further accelerate tumor progression [29].

## Discussion

While imaging-based ST provides unparalleled resolution for dissecting tissue heterogeneity, its utility is often constrained by limited gene recovery and imperfect cell segmentation. To address these challenges, we developed RedeFISH. First, RedeFISH leverages RL to frame cell segmentation as a strategic decision-making problem, iteratively optimizing expression profile similarity to overcome the reliance on staining images in supervised methods and transcript misassignment in unsupervised methods. Second, RedeFISH performs segmentation in a complete transcriptome-driven manner, ensuring optimal alignment of segmented cell expression profiles and enabling whole transcriptome recovery, thus alleviating the limitation of restricted gene detection in imaging-based ST. Third, RedeFISH exhibits broad compatibility across diverse imaging-based ST platforms and, with appropriate preprocessing, is also applicable to sequencing-based ST technologies. Across multiple datasets, RedeFISH yields cell expression profiles that more closely match single-cell references, achieving an average improvement of 9.7% in cosine similarity and a 13.9% reduction in RMSE compared with existing methods. It also improves cell boundary delineation, with an approximately 19% gain over current approaches. In terms of morphology, RedeFISH increases cell area and girth by over 20%, while maintaining roundness at a level comparable to SOTA methods, which may be attributed to its reliance on the spatial distribution of transcripts for morphological inference, introducing inherent uncertainty and stochasticity. By incorporating noise sampling, RedeFISH distinguishes intracellular from extracellular transcripts, accurately delineating cell boundaries in transcript-rich epithelial and immune cell-enriched regions, and achieving robust segmentation even in the highly challenging stromal-enriched areas. Collectively, these results demonstrate that RedeFISH effectively captures the structural organization of diverse tissues, underscoring its robustness for imaging-based ST cell segmentation. Furthermore, they highlight the considerable potential of RL in the analysis of spatial omics data, and we anticipate its broader application in addressing a wide range of challenges in this field.

Integrative analysis of imaging-based ST and scRNA-seq data is pivotal to elucidate spatiotemporal biological processes by resolving the spatial context of gene expression, enabling mechanistic insights into cell–cell interactions and microenvironmental regulation. The deployment of RedeFISH is projected to establish a new standard in spatial omics analysis by synergistically combining the high-plex capability of scRNA-seq with the spatial fidelity of imaging-based ST, ultimately generating biologically interpretable datasets where every measured transcript can be mapped within its native tissue microenvironment while maintaining whole transcriptome breadth. Our application of RedeFISH to mouse ileum dataset revealed a spatially organized *Wnt3*–*Fzd5* signaling axis between Paneth cells and intestinal stem cells, mechanistically linking niche topography to stem cell maintenance through ligand–receptor topographic mapping. In human breast cancer specimens, RedeFISH revealed pseudotemporal up-regulation of *MDM2* and *DCAF7* during DCIS-to-invasive transition, coupled with microenvironmental remodeling featuring myoepithelial cell loss and recruitment of macrophages.

Collectively, these results indicate that RedeFISH serves not only as a highly accurate cell segmentation tool but also, through its whole-transcriptome segmentation outputs, facilitates a broad spectrum of downstream analyses, thereby substantially enhancing the application and interpretability of ST data across diverse biomedical research contexts, ranging from fundamental studies of developmental biology to translational investigations in oncology.

## Methods

### Model overview

RedeFISH leverages a policy gradient-based deep RL framework to perform cell segmentation in imaging-based ST and scRNA-seq data by optimizing expression similarity between ST-derived segments and single-cell transcriptomes. The pipeline begins with data preprocessing to meet the input requirements of the neural network. A segmentation module and a deconvolution module are then implemented to assign transcripts and align single cells within segmented regions. RedeFISH iteratively alternates between these 2 modules over predefined epochs, producing a set of candidate cell segments. Finally, postprocessing refines the segmentation by defining polygonal cell boundaries and imputing whole-transcriptome expression profiles for the segmented cells.

### Preprocessing

For data preprocessing, RedeFISH first encodes all molecules into integers. Consider an imaging-based ST dataset containing *n* molecules with spatial coordinates {*x_i_*, *y_i_*} and associated genes {*g_i_*}, where *i* = 1,...,*n*, together with an scRNA-seq expression matrix *U* of dimensions *s* × *m*, representing *s* cells and *m* genes. We assign a unique identifier to each gene in the set *K*, which comprises *k* intersection genes {*k_i_*, *i* = 1,...,*k*} detected in both scRNA-seq and imaging-based ST. Accordingly, we use positive integers 1 to *k* to represent all genes in *K*. For ST data, each molecule is encoded using its corresponding identifier if *g_i_* ⊆ {*k_i_*}, and otherwise assigned the value *k* + 1. For scRNA-seq data, we construct a new expression matrix *S* of dimensions *s* × *k*, representing the *s* cells and *k* intersection genes.

Next, in order to calculate noise distance scores *NDS_i_ i* = 1,..,*n* for molecules, we implement a k-d tree structure using the Scipy package in Python to find top *d*th nearest neighbors for each molecule based on Euclidean distance metric. Then, for each molecule, mean distance *d_i_*, *i* = 1,..,*n* of the *d*th nearest neighbors are determined and the noise distance scores are calculated as:

$$\begin{array}{c} {\mathrm{NDS}}_{i}=\text{ReLU}\left[1-d_{i}/Q\left(d,\;q\right)\right]\;i=1,\ldots ,n;q\subset \left[0,1\right] \end{array}$$ (1)

where *Q* is quantile function and ReLU represents rectified linear unit activation function. This score measures the distance between a molecule and its *d*th nearest neighboring molecules. Hence, a molecule with a low distance score, which signifies a greater separation from other molecules, enhances the likelihood of being classified as background noise within the Segmentation Module.

### Segmentation Module

The primary objective of the Segmentation Module is to perform molecule-level classification, ultimately yielding cell regions and aggregating molecules with identical classifications to construct expression profiles. In practice, an ST dataset may contain hundreds of thousands of cells (denoted as *c*), making it impractical to perform *c*-classification for each molecule when *c* is extremely large. However, given a list of candidate cells with spatial locations {*X_i_*, *Y_i_*}, *i* = 1,...,*c*, RedeFISH identifies top *m*th nearest candidate cells and performs an *m*-classification for each molecule, where *m* is much smaller than *c*. The list of candidate cell locations could be estimated by RedeFISH (see “Estimate candidate cell locations”) or any other algorithm- or experiment-based methods, such as clustering or staining. These methods provide prior estimates of cell center locations, which are used for the initial iteration of RedeFISH. In addition, we define and randomly initialize a *p*-dimensional trainable vector *f* for each candidate cell, as well as a *p*-dimensional trainable vector *g* for each gene. For a pair of molecular *i* and one of its nearest candidate cell *j*, a location vector representing relative position is generated using Eq. (2).

$$\begin{array}{c} \begin{array}{c} h_{i,j}=\text{Linear}\left(\text{ReLU}\left(\text{Linear}\left(\text{Coordinate}\left(i\right)-\text{Coordinate}\left(j\right)\right)\;\right)\right)\\ i=1,\ldots ,n;j=1,\ldots ,m \end{array} \end{array}$$ (2)

The coordinate for candidate cell refers to coordinate of cell center. Integrating molecular coordinates with candidate cell coordinates enhances the likelihood of predicting adjacent molecules that belong to the same cells. Based on the vectors *f*, *g*, and *h*, RedeFISH generates a combined feature vector for each pair of molecular *i* and one of its corresponding candidate cell *j* through:

$$\begin{array}{c} \begin{array}{c} {\text{feature}}_{i,j}=\text{Dropout}\left[\text{LayerNorm}\left(f_{j}\right)+\text{LayerNorm}\left(g_{i}\right)+\text{LayerNorm}\left(h_{i,j}\right)\right] \end{array} \end{array}$$ (3)

$$\begin{array}{c} {\text{feature}}_{i,j}=\text{Linear}\left(\text{ReLU}\left(\text{Linear}\left(\text{ReLU}\left(\text{Linear}\left({\text{feature}}_{i,j}\right)\right)\right)\right)\right) \end{array}$$ (4)

For molecular *i* and all its candidate cells, the softmax operation is applied to calculate the assignment probabilities of molecule *i* to its *m*th nearest cells.

$$C\_{\text{prob}}_{i}=\text{Dropout}\left[\text{Softmax}\left({\text{feature}}_{i}\right)\;\right]$$ (5)

Here, categorical distribution *Cat*(*C_prob_i,1_*, *C_prob_i,2_*, ..., *C_prob_i,m_*) is used to model the assignment probabilities of molecule *i* to its *m*th nearest cells. To determine background noise molecule, a trainable noise feature matrix with *c* × *2* dimensions is initialized in Eq. (6), followed by a softmax operation to calculate the probabilities for a binary noise classification.

$$\begin{array}{c} {\text{noise}}_{i}=\text{Concat}\left(1-{\mathrm{NDS}}_{i},\;{\mathrm{NDS}}_{i}\right)\;i=1,\ldots ,n \end{array}$$ (6)

$$N\_{\text{prob}}_{i}=\text{Softmax}\left({\text{noise}}_{i}\right)\;$$ (7)

Similarly, RedeFISH employs a categorical distribution *Cat*(*N_prob*_*i* ,1_*, N_prob*_*i*,2_) to model the assignment probabilities of molecule *i* to either noise or non-noise categories. Based on these categorical distributions, molecule *i* was assigned to one of its nearest cells (*G_i_*) and classified as either noise or non-noise (*H_i_*). In addition, RedeFISH estimates the coordinates of cell centers by averaging the coordinates of the corresponding molecules, and then calculates distance *g_i_* between molecular *i* and corresponding cell center. Hence, in each batch, center distance score *CDS_i_* for molecular *i* was calculated by*:*

$$\begin{array}{c} {\mathrm{CDS}}_{i}=\text{ReLU}\left[1-g_{i}/Q\left(g,\;q\right)\right]\;i=1,\ldots ,b;q\subset \left[0,\;1\right] \end{array}$$ (8)

where *Q* is the quantile function of *d*. Then, reward for assigning molecule *i* to cell *j* in epoch *e* is given as:

$${\text{reward}}_{i}=\left\{\begin{array}{l} {\mathrm{NDS}}_{i}\times {\mathrm{CDS}}_{i}\times {\mathrm{ESS}}_{i}\;\text{if}\;i\in \left\{\text{noise}\right\}\\ \left(1-{\mathrm{NDS}}_{i}\right)\times \mathrm{CDS}\times {\mathrm{ESS}}_{i}\;\text{otherwise}\; \end{array}\right.$$ (9)

The variable *ESS* represents the expression similarity scores, which will be elaborated upon in the next section. The *NDS* quantifies the likelihood of a molecule being assigned to noise categories, while the *CDS* measures the distance from the molecule to the corresponding cell center. Based on formula of policy gradient, we define loss function for Segmentation Module as:

$$\begin{array}{c} \text{region}\_\text{loss}=-\frac{1}{n}\sum_{i}{\text{reward}}_{i}\times \left[\ln P\left(G_{i}|C\_{\text{prob}}_{i}\right)+\ln P\left(H_{i}|N\_{\text{prob}}_{i}\right)\right] \end{array}$$ (10)

### Deconvolution Module

The primary objective of the Deconvolution Module is to improve the expression profiles of segmented cells by maximizing the expression similarities between scRNA-seq data and the profiles generated by the Segmentation Module. In addition, this module generates expression similarities (*ES*) that are utilized in the Segmentation Module to calculate rewards. Given the scRNA-seq expression matrix *S* with *t* distinct cell types, RedeFISH generates a sub-expression matrix *S_i_* (*i* = 1,...,*t*) with dimension of *ni* × *k* for each cell type, where *ni* represents the number of single cells for cell type *i*. In addition, we introduce a trainable weight variable *W_i_,* having dimensions of *cs* × *ni*, where *cs* represents the predefined number of cell states. Then, expression matrix *S_i_′* for cell type *i* with dimensions *cs* × *k* was generated using Eq. (11).

$$S_{i}^{\prime }=\text{Dropout}\left[\text{SoftPlus}\left(W_{i}\right)\times S_{i}\right]$$ (11)

where SoftPlus refers to smoothed ReLU activation function. By concatenating the generated expression matrices at the cell type level, we obtained expression matrix *S*′ with dimensions (*cs* × *t*) × *k.*

$$S^{\prime }=\text{Concat}\left(\left[S_{1}^{\prime },S_{2}^{\prime },\ldots ,S_{t}^{\prime }\right]\right)$$ (12)

In practice, employing *S′* for cell segmentation not only conserves memory but also improves efficiency, as (*cs* × *t*) is typically significantly smaller than the total number of single cells. Next, we introduce another trainable weight variable *V* with dimensions of *c* × (*cs* × *t*), and this variable is applied to calculate predicted expression profiles *PE*, which is a *c* × *k* matrix:

$$\mathrm{PE}=\text{Dropout}\left[\text{SoftPlus}\left(V\right)\right]\times S^{\prime }$$ (13)

Thus, for a candidate cell *j*, 2 expression profiles are generated: One, denoted as *PE_j_,* is derived from the scRNA-seq reference via Eq. (13); the other, denoted as *E_j_*, is obtained within the Segmentation Module through transcript assignment. Based on the 2 profiles, expression similarity (*ES*) and expression residual (*ER*) are determined as:

$${\mathrm{ES}}_{j}=\text{CosineSimilarity}\left({\mathrm{PE}}_{j},\;E_{j}\right)$$ (14)

$${\mathrm{ER}}_{j}={\mathrm{PE}}_{j}-E_{j}$$ (15)

$$\begin{array}{c} {\mathrm{ER}}_{j}=\left[{\mathrm{ER}}_{j}-\min\left({\mathrm{ER}}_{j}\right)\right]/\left[\max\left({\mathrm{ER}}_{j}\right)-\min\left({\mathrm{ER}}_{j}\right)\right] \end{array}$$ (16)

To maximize expression similarities between the 2 predicted expression profiles, RedeFISH minimizes loss function given in Eq. (17).

$$\text{alignment}\_\text{loss}=\frac{1}{c}\sum_{j}\left(1-{\mathrm{ES}}_{j}\right)$$ (17)

Suppose molecular *i* is assigned to cell *j* in the Segmentation Module, and molecular *i* belongs to a gene that encoded as *k*. The expression similarity score (*ESS_i_*) for this molecular is given in Eq. (18).

$${\mathrm{ESS}}_{i}=\left\{\begin{array}{l} {\mathrm{ER}}_{j,k}\;\;\text{if}\;i\in \left\{\text{noise}\right\}\\ \left(1-{\mathrm{ER}}_{j,k}\right)\;\text{otherwise}\; \end{array}\right.$$ (18)

This expression similarity score is incorporated into Eq. (9) of the Segmentation Module to compute the rewards.

### Postprocessing

Following the final assignment of molecules and the determination of cellular expression profiles, postprocessing is performed to delineate polygonal cell boundaries and impute whole-transcriptome expression profiles for the identified cells. RedeFISH first utilizes the alphashape algorithm in Python to determine polygonal regions of cell bodies, followed by the refinement of molecular assignments based on the results of the alphashape analysis to generate final expression profiles. Then, a matrix *A* was computed to represent the pairwise cosine similarity of log expression between cells in the scRNA-seq data and the output of RedeFISH. For each cell in the output, RedeFISH identifies the *r*th most similar cells based on matrix *A*. Additionally, for each cell in the scRNA-seq dataset, RedeFISH calculates the occurrence of single cells (*O*) that are present among the *r*th most similar cells. Assuming that there are *n* cells in the RedeFISH output, the occurrence scores (*OS*) for single cell *j* are defined in Eq. (19).

$${\mathrm{OS}}_{j}=\log\left(n/O_{j}\right)$$ (19)

For cell *i* in the RedeFISH output, we calculate chosen weights for the *r*th most similar single cells by multiplying the expression similarity with corresponding occurrence scores. Thus, we generate a new matrix *W* with *n* × *r* dimensions by sorting the chosen weights in descending order for each cell. Finally, we impute the whole-transcriptome expression profile for each cell by performing a weighted average of the expression profiles, utilizing the top *k* single cells and their corresponding weights in *W*.

### Model implement

At the beginning, RedeFISH preforms data preprocessing (see “Preprocessing”) to encode molecules in ST data and generate expression matrix of intersection genes for scRNA-seq data. To initialize the 2 modules, RedeFISH assigns molecules to their nearest candidate cells and generates an expression matrix for the initial assignments and the initial values of *ER*, *ES*, and *ESS* are then determined. After that, the model undergoes iteration for a predetermined number of epochs. During each epoch, RedeFISH first employs a batch training strategy to compute the rewards for each molecule, followed by the minimization of region loss based on these rewards. This results in the acquisition of a cell segmentation outcome for each epoch within the Segmentation Module. Next, in Deconvolution Module, RedeFISH updates *ESS* and minimize alignment loss. The *ESS* obtained from the previous epoch is utilized to compute rewards in the Segmentation Module for the next epoch. After completing all epochs, RedeFISH carries out an additional Segmentation Module to determine the final molecular assignments. In this scenario, dropout layers are omitted, and molecular assignments and noise classifications are determined by selecting the maximum probabilities within categorical distributions, rather than employing a sampling approach. In the end, RedeFISH conducts the postprocessing (see the previous section) for generating polygonal regions and imputing whole-transcriptome expression profiles.

To train the RedeFISH model, Adam is selected as the optimizer and exponential learning rate schedule is applied with a multiplicative factor decay of 0.996 for one epoch. The number of epochs was set to a default of 500, with the initial learning rates established at 0.01 for the noise feature matrix and 0.002 for other variables, respectively. In preprocessing, *d* and *q* are set to 100 and 0.999, respectively, for calculating the distance scores. In Segmentation Module, we set *m* = 8 for cell assignments and *p* = 20 for dimensions of cell features and gene features. Additionally, batch size is set to the number of molecules divided by 50 with a maximum value of 2,000,000. In Deconvolution Module, we set *cs* = 10 and the number of iterations in each epoch to 30. In postprocessing, *r* is set to 40 and *k* is set to 20 for imputation of expression profiles. The manuscript consistently employs these default parameters across all experiments, unless otherwise specified in particular notifications.

### Estimate candidate cell locations

The purpose of the estimate candidate cell locations step is to generate a set of prior candidate cell center locations for the initial iteration of RedeFISH. To achieve this, we utilize a 3-step approaches, including (a) identifying marker genes based on scRNA-seq data, (b) performing clustering of marker genes based on spatial distribution of corresponding molecules, and (c) clustering molecules into candidate cells for each category of marker gene.

We first identify marker genes for each cell type in the normalized scRNA-seq data. For gene *i* within cell type *j*, the average expression levels (*A_ij_*) are calculated and the percentage expression (*P_ij_*) is determined by dividing the average expression by the total average expression of that gene. Thus, we generate score matrix (*S_ij_*) for genes and cell types using Eq. (20).

$$S_{i,j}=\log\left(A_{i,j}+1\right)\times P_{i,j}$$ (20)

The top *p* marker genes are selected for each cell type based on the descending order of corresponding columns in the score matrix. By eliminating duplicate marker genes, we ultimately obtained a list of *m* unique marker genes.

Next, we perform clustering based on the spatial distribution of the unique marker genes. Given a prior cell radius *r*, a grid is generated with a spacing of 2*r* and a total number of *n* nodes. We then calculate *m*-dimensional vectors by counting the marker genes located within a radius of $2r/\sqrt{\pi }$ adjacent to the nodes to represent features of the nodes, thereby generating a feature matrix *F* with dimension of *m* × *n*. The feature matrix is further standardized by columns. In addition, an *m* × *m* dimensional similarity matrix *s* is generated by calculating pairwise Pearson’s correlation coefficient between each pair of rows in *F*. Based on the similarity matrix, we employ AgglomerativeClustering function in sklearn to cluster *m* unique marker genes into *c* categories. For each category, we generate a threshold expression vector *t* by selecting the maximum expression value of the corresponding gene in *A* and summing these values.

To generate candidate cell locations, we employ the DBSCAN and *K*-means algorithms for clustering molecules within each category, and subsequently apply the Mean Shift algorithm to integrate the category-level outcomes and derive the final candidate cells. In detail, we first ascertain the total number of corresponding molecules *h* located in the adjacent area of each node for each category. After that, we define valid nodes by determining whether *h* exceeds the cutoff established in Eqs. (21) and (22).

$$\text{cutoff}=Q\left(h,\;q\right)\times 0.01\;$$ (21)

$$\text{cutoff}=\text{Mean}\left[h\left(h>\text{cutoff}\right)\right]$$ (22)

Hence, the total number of valid nodes *v* is determined. Next, DBSCAN function in sklearn is applied for unsupervised clustering molecules into categories with parameter eps = 2*r*. Based on the outcomes, *K*-means is then employed to cluster the molecules in each category into candidate cell and to generate candidate cell locations by averaging the coordinate of molecules for each candidate cell. For the marker gene category *c*, the *k* number in *K*-means algorithm is defined in Eq. (23).

$$k=\mathrm{int}\left[0.25h\times \log\left(\mathrm{Max}\left(t\right)/t_{c}+1\right)\right]$$ (23)

After the clustering of molecules into candidate cells in all marker gene categories, the MeanShift function with a bandwidth parameter set to *r*/5 in sklearn is employed to merge spatially adjacent candidate cells and produce the final candidate cell locations.

To implement the candidate cell location estimation process, the parameter *p* for number of marker genes for each cell type is set to 3 and the parameter *c* for the number of marker gene categories is set to 10. Meanwhile, *q* in equation is set to 0.9. The parameter *r* for the prior cell radius is determined based on the resolution of ST and is typically maintained at 6.5 μm.

### RedeFISH with nuclear boundary priors

Current imaging-based ST platforms routinely provide nuclear segmentation results. RedeFISH is naturally suited for inferring cell boundaries based on these nuclear segmentations. During the RL stage, the distance from transcripts to the cell center in Eq. (2) is replaced with the distance from transcripts to the nuclear region, with distances set to zero for transcripts located inside the nucleus. In the postprocessing stage, after obtaining cell boundaries using alphashapes as described in the “Postprocessing” section, any regions overlapping with other nuclear regions are removed. The resulting cell boundaries are then combined with the prior nuclear boundaries to derive the final cell regions and corresponding expression matrices. Gene imputation is subsequently performed following the procedure described in the “Postprocessing” section.

### Datasets for benchmarking

#### Human non-small cell lung cancer

ST dataset of human NSCLC from CosMx platform is available at https://nanostring.com/products/cosmx-spatial-molecular-imager/nsclc-ffpe-dataset/ [4]. We chose Lung5_Rep1 in this study, including 37,226,610 transcripts for 980 genes. Meanwhile, an h5ad file containing annotation information pertaining to scRNA-seq dataset is accessible at https://cellxgene.cziscience.com/collections/edb893ee-4066-4128-9aec-5eb2b03f8287 [30].

#### Human breast cancer

Replicate 1 of human breast cancer ST dataset is available at https://www.10xgenomics.com/products/xenium-in-situ/preview-dataset-human-breast based on Xenium platform [5], including 43,664,530 transcripts for 541 genes. In addition, scFFPE-seq dataset from the same sources was applied for RedeFISH algorithm. We used the clustering result to perform RedeFISH, since annotation is not available.

#### Mouse ileum

The MERFISH-based mouse ileum dataset is available at https://doi.org/10.5061/dryad.jm63xsjb2 [11] composed of 819,665 transcripts for 241 genes. We additionally downloaded scRNA-seq dataset at https://singlecell.broadinstitute.org/single_cell with an accession of SCP1038 [31]. Moreover, cell annotation information was extracted from corresponding metadata file.

#### *Mouse bra*in *MERSCOPE*

We downloaded sample S1R1 MERSCOPE dataset of mouse brain from Vizgen website (https://vizgen.com/data-release-program/), including 54,712,414 for 649 genes. In addition, we procured scRNA-seq dataset and corresponding metadata from Allen Brain Atlas (https://portal.brain-map.org/atlases-and-data/rnaseq/mouse-whole-cortex-and-hippocampus-10x) [32].

#### Mouse liver

Sample L1R2 MERSCOPE dataset of mouse liver is available at https://vizgen.com/data-release-program/ composed of 225,890,863 transcripts for 385 genes. We obtained droplet scRNA-seq dataset with accompanying annotations from https://figshare.com/articles/dataset/Processed_files_to_use_with_scanpy_/8273102/2 [33].

#### Mouse brain Stereo-seq

The Stereo-seq-based mouse brain ST dataset was downloaded from MOSTA website (https://db.cngb.org/stomics/mosta/download/) [34]. The scRNA-seq dataset is consistent with those employed in the Mouse Brain MERSCOPE dataset.

#### Human lung cancer

The human lung cancer Xenium dataset is available at https://www.10xgenomics.com/cn/datasets/preview-data-ffpe-human-lung-cancer-with-xenium-multimodal-cell-segmentation-1-standard. We used the same single-cell reference as that employed for the Human Non-small Cell Lung Cancer dataset.

### Implement RedeFISH on datasets

We first processed the scRNA-seq data acquired from various sources. For Human-NSCLC dataset, we used the annotation from “cell_type_major” in the h5ad file that includes 24 major cell types. For Human Breast Cancer dataset, we labeled each cluster in scFFPE-seq data with a corresponding cell type designation (Table S1). For Mouse Ileum dataset, we reclassified 3 subclasses of transit-amplifying (TA) cells (“TA_3”, “TA_4”, and “TA_5”) as enterocyte cells due to their high expression levels of *Slc51a* and *Slc5a1*, which are established markers for enterocyte cells.

Next, we estimated candidate cell locations for each dataset using the approaches in “Estimate candidate cell locations”. Specifically, the prior cell radius *r* was set to 36, 6.5, 30, 6.5, 13, and 6.5 for Human-NSCLC (CosMx), Human Breast Cancer (Xenium), Mouse Ileum (MERFISH), Mouse Brain (MERSCOPE), Mouse Brain (Stereo-seq), and Mouse Liver (MERSCOPE) datasets, respectively. In addition, we set the number of marker gene categories *c* to 4 for Mouse Brain (MERSCOPE) and Mouse Brain (Stereo-seq) datasets. Finally, based on the estimated candidate cells, RedeFISH was performed for cell segmentation and gene imputation using the same radius (*r*) as defined above and other default parameters.

The Human Lung Cancer (Xenium) dataset includes GT cell boundaries. Following a strategy similar to that used in Proseg, we used the Xenium output as prior information. Specifically, the nuclear boundaries provided by Xenium were used as priors, and cell boundaries were inferred using the procedure described in the “RedeFISH with Nuclear Boundary Priors” section. The cell radius parameter *r* was set to 6.5, with all other parameters kept at their default settings.

By exception, a different preprocessing procedure was performed for the Mouse Brain (Stereo-seq) dataset, which is based on sequencing-based ST platforms. We initially derived a list of molecules with spatial coordinates by transforming the spot-by-gene expression matrix into an imaging-based format, wherein each sequencing-based unique molecular identifier (UMI) was treated as a discrete molecule. Then, top 2000 intersection genes, exhibiting the highest levels of expression in ST data, were identified and subsequently encoded as positive integers from 1 to 2000, whereas other genes were encoded as 2001. After that, candidate cell estimation and RedeFISH were performed, with the quantile *q* set to 0.9999 in RedeFISH.

### Implement Baysor on datasets

We implemented Baysor v0.5.0 [11] on the benchmarking datasets. Firstly, we used “--save-polygons GeoJSON” to generate polygonal regions of segmented cells. Then, the scale parameter was set to 50, 7, 6.5, 30, and 9 for Human-NSCLC (CosMx), Human Breast Cancer (Xenium), Human Lung Cancer (Xenium), Mouse Ileum (MERFISH), and Mouse Brain (MERSCOPE) datasets, respectively, to maintain an approximately equivalent cellular radius between segmented cells from Baysor or original publication. In addition, the implementation of Baysor is infeasible for ultra-large datasets, such as Mouse Liver and Mouse Brain (Stereo-seq) datasets, due to memory limitation (exceed 5 TB).

### Implement Proseg on datasets

We analyzed the Human-NSCLC (CosMx), Human Breast Cancer (Xenium), Mouse Liver (MERSCOPE), Human Lung Cancer (Xenium), and Mouse Brain (MERSCOPE) datasets using Proseg v2.0.4 [13]. The preliminary transcript assignment results and transcript information provided with these datasets were input into Proseg, and analyses were performed with default parameters. The Mouse Ileum (MERFISH) dataset was excluded because the preliminary transcript assignment results were not available, and the Mouse Brain (Stereo-seq) dataset was not included as it is a sequencing-based ST platform.

### Implement Cellpose-SAM on datasets

We used the Cellpose package (v4.0.8) in Python, which implements the Cellpose-SAM model [17], to analyze the Human Breast Cancer (Xenium), Mouse Brain (MERSCOPE), and Human Lung Cancer (Xenium) datasets. For the Human Breast Cancer (Xenium) dataset, H&E-stained images were used as input to obtain nuclear mask matrices. For the Mouse Brain (MERSCOPE) and Human Lung Cancer (Xenium) datasets, 4′,6-diamidino-2-phenylindole (DAPI)-stained images were used as input to generate nuclear mask matrices. The nuclear boundaries were then expanded outward by up to 5 μm or until reaching the boundary of neighboring cells to derive the cell mask matrices. Based on these masks, polygon coordinates of cell boundaries and corresponding cellular expression profiles were obtained. All parameters were kept at their default settings.

### Implement Stardist on datasets

We used StarDist (v0.9.2) to analyze the Human Breast Cancer (Xenium), Mouse Brain (MERSCOPE), and Human Lung Cancer (Xenium) datasets. The input data and postprocessing steps were identical to those used for Cellpose-SAM, and all parameters were kept at their default settings.

### Expression similarity

As one goal of RedeFISH is to optimize expression profiles of segmented cells, we measured expression similarity between segmented cells and single cells in scRNA-seq data. To achieve this, cosine similarity and root mean square error (RMSE) were calculated as evaluation metrics for assessing the similarities. We firstly identified the intersection genes between ST and scRNA-seq data, subsequently generating expression matrices *A* and *B* based on the shared genes. The total UMI counts of each cell in *A* and *B* were additionally normalized to the total number of the intersection genes. Thus, given a segmented cell *i* and single cell *j*, the corresponding cosine similarity (*SIM_COS*_*i*,*j*_) and RMSE (*SIM_RMSE*_*i*,*j*_) were computed as:

$$\begin{array}{c} \mathrm{SIM}\_{\mathrm{COS}}_{i,j}=\text{CosineSimilarity}\left(A_{i},\;B_{j}\right) \end{array}$$ (24)

$$\mathrm{SIM}\_{\text{RMSE}}_{i,j}=\sqrt{\frac{1}{k}\sum {\left(A_{i}-B_{j}\right)}^{2}}$$ (25)

where *k* refers to number of the intersection genes. For each segmented cell *i*, we generated 2 lists of similarities between *i* and all single cells, therefore {*SIM_COS*_*i*,1_, ..., *SIM_COS*_*i*,*n*_} and {*SIM_RMSE*_*i*,1_,...,*SIM_RMSE*_*i*,*n*_}. Finally, overall similarities *SIM_C_i_* and *SIM_R_i_* were calculated by averaging the top 20 highest values from the first list and the top 20 lowest values from the second list, respectively. We compared *SIM_C* and *SIM_R* for all identified cells across the 3 approaches using boxplot and the Wilcoxon test across 6 datasets. A higher *SIM_C* and a lower *SIM_R* indicate an enhanced level of expression similarity between segmented cells and single cells. The corresponding scRNA-seq data used for similarity calculations in this section are provided in the “Datasets for benchmarking” section.

### Boundary alignment

To assess the agreement between segmented cells and GT boundaries, we evaluated both sensitivity and specificity on Human Lung Cancer (Xenium) dataset. For sensitivity, each GT cell was matched to its nearest segmented cell and their regional overlap was measured. For specificity, each segmented cell was matched to its nearest GT cell. Agreement between regions was quantified using the IoU, defined as the ratio of the intersection area to the union area. To estimate cell morphological similarity, we firstly used the Python package Shapely (v2.1.2) to compute the area *s* and girth *c* of each segmented cell, and calculated roundness *R* according to the following formula:

$$R=4\pi s/c^{2}$$ (26)

Then, we plotted the density curves of morphological features for segmented cells from each method and the GT cells, and computed the IoU between the areas under the corresponding curves to assess the consistency of cell morphology.

### Imputation accuracy

To assess imputation accuracy, 30% of genes were randomly masked in the Human-NSCLC (CosMx), Human Breast Cancer (Xenium), Mouse Liver (MERSCOPE), Mouse Brain (MERSCOPE), and Mouse Ileum (MERFISH) datasets. RedeFISH was applied using the remaining 70% of genes to perform cell segmentation and gene expression imputation. The similarity between the imputed expression and the original expression of the masked genes was then computed as described in the “Expression similarity” section. This procedure was repeated 3 times for each dataset.

### Evaluation of the impact of the noise sampling step

To evaluate the impact of noise sampling on cell segmentation in RedeFISH, we simulated a variant without noise sampling by setting the probability of assigning molecules to non-noise categories in Eq. (7) to a constant value of 1, and fixing the *NDS* score in Eq. (9) to 1, while keeping all other components of RedeFISH unchanged. We then ran this modified version on the Human Lung Cancer (Xenium) dataset and assessed its effect on cell segmentation from both cellular region and morphological perspectives. Specifically, following the procedure described in “Boundary alignment,” we computed the relevant metrics and compared the statistical results with and without the noise sampling step.

### Runtime efficiency

We compared the runtime efficiency of RedeFISH, Baysor, and Proseg on the Human-NSCLC (CosMx), Human Breast Cancer (Xenium), Mouse Liver (MERSCOPE), Mouse Brain (MERSCOPE), Mouse Ileum (MERFISH), and Human Lung Cancer (Xenium) datasets. RedeFISH was executed using a single NVIDIA RTX 5880 Ada GPU. For Baysor and Proseg, which are central processing unit (CPU)-based methods, the maximum number of threads was set to 16.

### Label transfer

To facilitate comparison of cell type distributions, we applied label transfer to automatically assign cell types from scRNA-seq data to segmented cells. For each cell, the top 20 scRNA-seq cells with the highest cosine similarity in expression profiles were identified. The similarity scores were then aggregated by cell type and normalized by the total similarity across the 20 cells, yielding a probability distribution of cell type assignments. The cell type with the highest probability was designated as the final identity of each cell.

We performed label transfer on the cell segmentation results obtained from RedeFISH, Baysor, Proseg, and the original studies to automatically assign cell types and to compare differences in cell type distributions across approaches.

### Analysis of mouse ileum dataset

We applied Leiden for cell clustering based on the expression profiles of identified cells, followed by manual cell annotation using marker genes. Then, t-distributed stochastic neighbor embedding (tSNE) in Scanpy packages was applied for cell type illustration. Recently, numerous algorithms have been developed for analyzing cell–cell interactions [35,36]. We employed CellPhoneDB to assess the significance of interactions between *Wnt* and *Fzd–Lrp* complexes.

### Analysis of human breast cancer dataset

Initially, Leiden combined with manual annotation was employed for the purpose of cell type annotation. Based on the spatial distribution of cell types, 4 ROIs were selected for downstream analysis, namely, DCIS #1, DCIS #2, DCIS #3, and invasive tumor. To investigate up-regulated genes in ROIs, we randomly selected 300 cells from one ROI and 100 cells from each of the remaining 3 ROIs, which was designated as the control group. Then, edgeR [37] was applied to identify up-regulated genes and compute the level of statistical significance. In addition, we used GEPIA [22] to analyze survival rate for several genes. In this analysis, we chose BRCA HER2^+^ nonluminal dataset, because the tissue block was annotated as HER2^+^/ER^+^/PR^−^, and set 60% and 40% for parameter Cutoff-High and Cutoff-Low, respectively. For pseudotime analysis, we randomly chose one start cell and 3 terminal cells from DCIS #1 and invasive tumor ROIs, respectively, and applied Palantir [25] with default setting for estimating pseudotime for DCIS and invasive tumor cells. We further employed a smooth spline method to perform curve fitting between pseudotime and gene expressions or cell type proportions.

To investigate cell type proportions in tumor microenvironment, for each ROI, we first generated a spatial coordinate list corresponding to the cell types of interest within that ROI. For example, in the DCIS #1 ROI, this corresponds to a list of spatial coordinates for DCIS #1 cells. Subsequently, we identified all cells located within a specified radius (10, 20, 50, and 100 μm) of any cell in the spatial coordinate list and calculated the cell type proportions based on the identified cells.

### AI-assisted graphical elements generation

Artificial intelligence (AI)-assisted tools (Doubao AI) were used to generate certain graphical elements in Fig. 1B. The authors reviewed and edited all outputs.

## Data Availability

Human-NSCLC. The ST dataset is available at https://nanostring.com/products/cosmx-spatial-molecular-imager/nsclc-ffpe-dataset/. The scRNA-seq dataset with annotation is available at https://cellxgene.cziscience.com/collections/edb893ee-4066-4128-9aec-5eb2b03f8287. Human Breast Cancer. Both ST and scFFPE-seq dataset of human breast cancer are available at https://www.10xgenomics.com/products/xenium-in-situ/preview-dataset-human-breast. Mouse Ileum. The mouse ileum ST dataset is available at https://doi.org/10.5061/dryad.jm63xsjb2. The scRNA-data are available at https://singlecell.broadinstitute.org/single_cell with an accession of SCP1038. Mouse Brain (MERSCOPE). The mouse brain (MERSCOPE) dataset is available at https://vizgen.com/data-release-program/. The scRNA-seq dataset and corresponding metadata are available on Allen Brain Atlas (https://portal.brain-map.org/atlases-and-data/rnaseq/mouse-whole-cortex-and-hippocampus-10x). Mouse Liver. The mouse liver (MERSCOPE) dataset is available at https://vizgen.com/data-release-program/. The droplet scRNA-seq dataset with annotation is available at https://figshare.com/articles/dataset/Processed_files_to_use_with_scanpy_/8273102/2. Mouse Brain (Stereo-seq). The Mouse Brain (Stereo-seq) dataset is available on MOSTA website (https://db.cngb.org/stomics/mosta/download/). The scRNA-seq dataset and corresponding metadata are available on Allen Brain Atlas (https://portal.brain-map.org/atlases-and-data/rnaseq/mouse-whole-cortex-and-hippocampus-10x). Human Lung Cancer. ST dataset is available at https://www.10xgenomics.com/cn/datasets/preview-data-ffpe-human-lung-cancer-with-xenium-multimodal-cell-segmentation-1-standard. The scRNA-seq dataset with annotation is available at https://cellxgene.cziscience.com/collections/edb893ee-4066-4128-9aec-5eb2b03f8287. The package of RedeFISH is available on github with detailed documentation (https://github.com/Roshan1992/RedeFISH).
