# Supplementary material for: Cell Segmentation as Strategic Decision Making
Source: Research (Wash D C). 2026 Jun 1;9:1304. doi: 10.34133/research.1304 (PMC13223358; doi:10.34133/research.1304)
Supplement: Supplementary 1 — Figs. S1 to S10 Table S1 [file research.1304.f1.zip › Supp Fig 2.pdf]

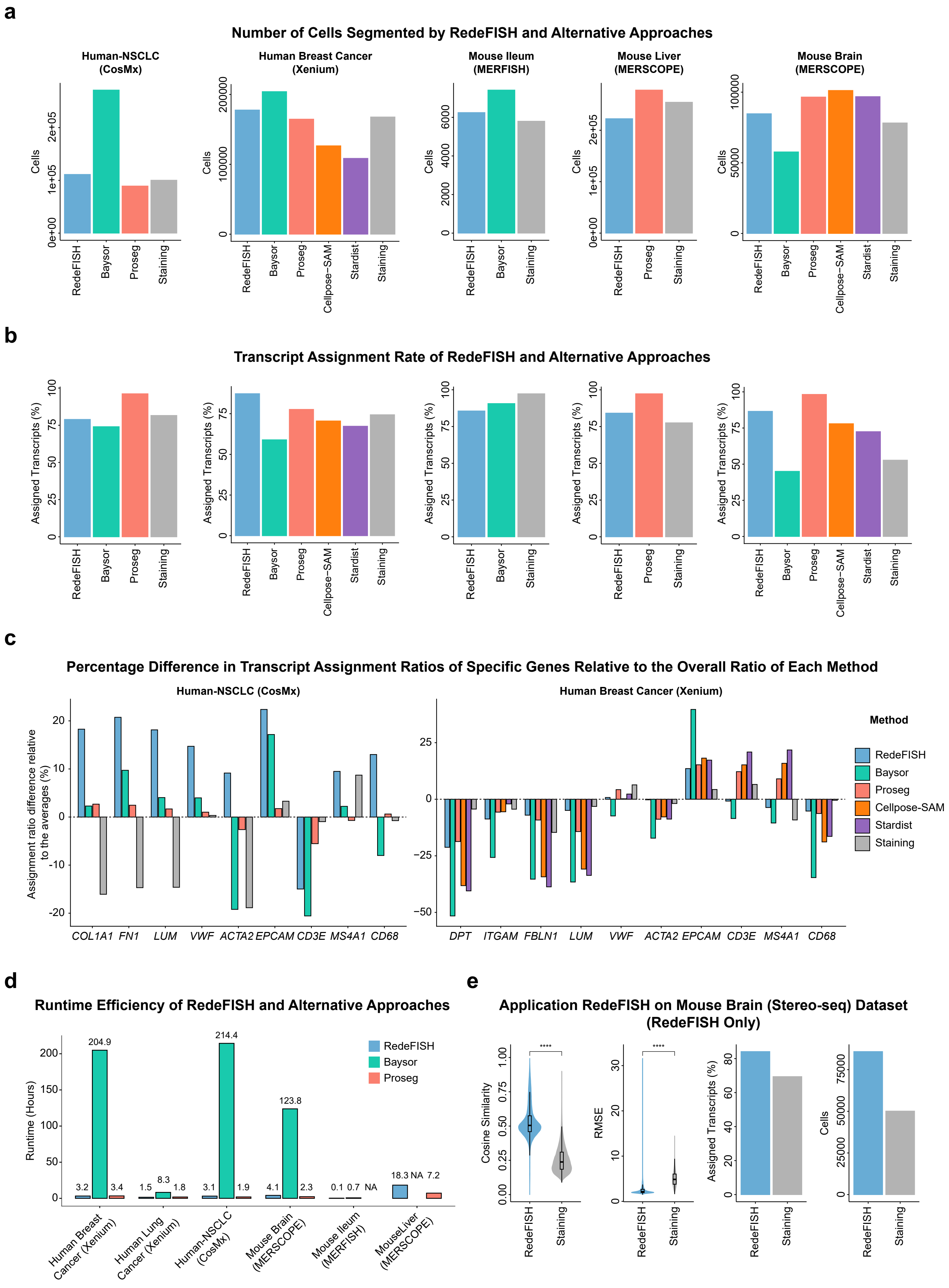

a
Number of Cells Segmented by RedeFISH and Alternative Approaches
Human-NSCLC (CosMx)
Human Breast Cancer (Xenium)
Mouse Ileum (MERFISH)
Mouse Liver (MERSCOPE)
Mouse Brain (MERSCOPE)
Cells
0e+00
1e+05
2e+05
0
100000
200000
2000
4000
6000
50000
RedeFISH
Baysor
Proseg
Cellpose−SAM
Stardist
Staining
b
Transcript Assignment Rate of RedeFISH and Alternative Approaches
Assigned Transcripts (%)
0
25
50
75
100
c
Percentage Difference in Transcript Assignment Ratios of Specific Genes Relative to the Overall Ratio of Each Method
Human-NSCLC (CosMx)
Human Breast Cancer (Xenium)
Assignment ratio difference relative to the averages (%)
COL1A1 FN1 LUM VWF ACTA2 EPCAM CD3E MS4A1 CD68
DPT ITGAM FBLN1 LUM VWF ACTA2 EPCAM CD3E MS4A1 CD68
Method
RedeFISH
Baysor
Proseg
Cellpose-SAM
Stardist
Staining
d
Runtime Efficiency of RedeFISH and Alternative Approaches
Runtime (Hours)
3.2
204.9
3.4
1.5
8.3
1.8
3.1
214.4
1.9
4.1
123.8
2.3
0.1
0.7
NA
18.3
NA
7.2
RedeFISH
Baysor
Proseg
Human Breast Cancer (Xenium)
Human Lung Cancer (Xenium)
Human-NSCLC (CosMx)
Mouse Brain (MERSCOPE)
Mouse Ileum (MERFISH)
MouseLiver (MERSCOPE)
e
Application RedeFISH on Mouse Brain (Stereo-seq) Dataset (RedeFISH Only)
Cosine Similarity
RMSE
Assigned Transcripts (%)
Cells
****
RedeFISH
Staining
