# Supplementary material for: Cell Segmentation as Strategic Decision Making
Source: Research (Wash D C). 2026 Jun 1;9:1304. doi: 10.34133/research.1304 (PMC13223358; doi:10.34133/research.1304)
Supplement: Supplementary 1 — Figs. S1 to S10 Table S1 [file research.1304.f1.zip › Supp Fig 3.pdf]

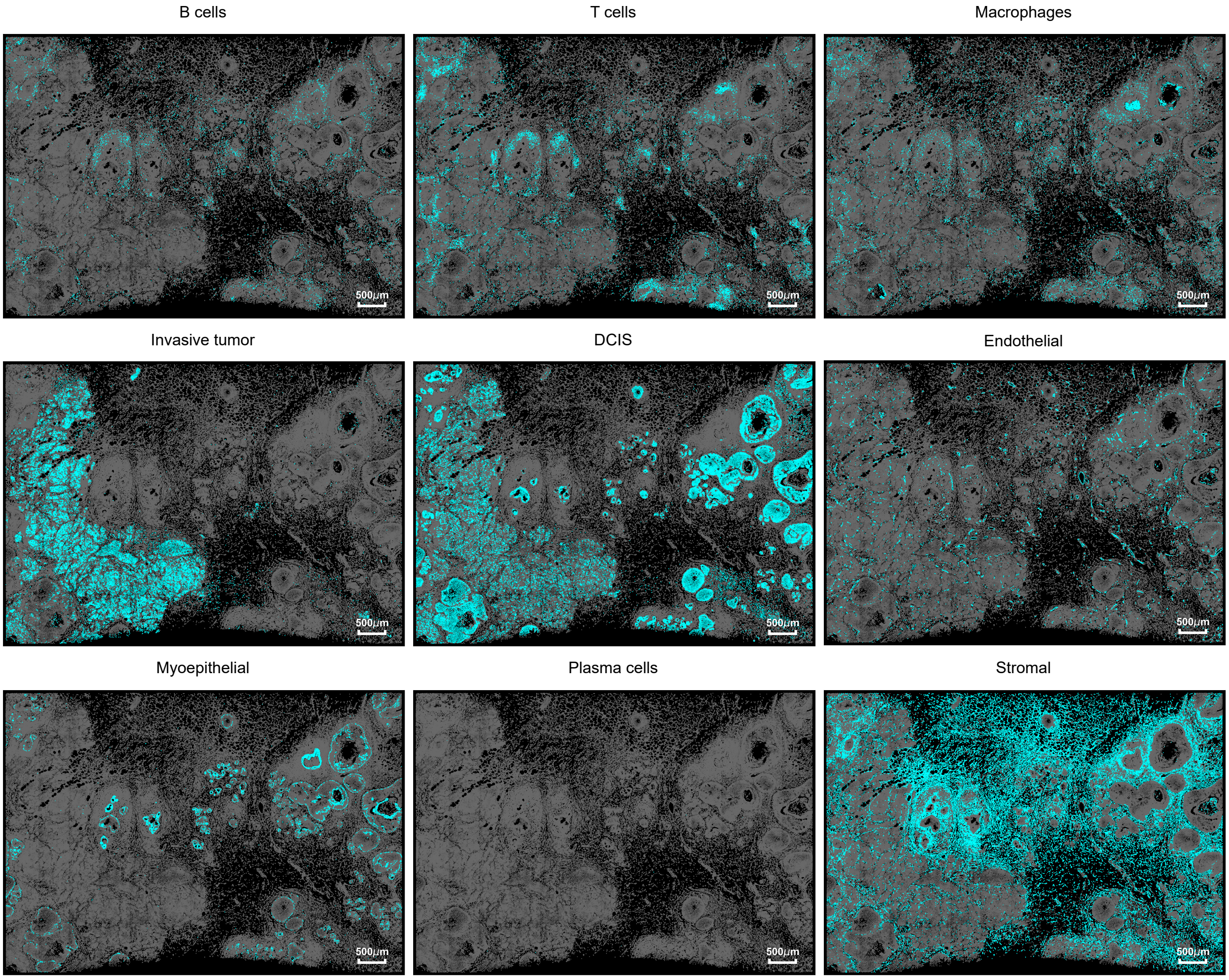
B cells
T cells
Macrophages
Invasive tumor
DCIS
Endothelial
Myoepithelial
Plasma cells
Stromal
500μm
