# Supplementary material for: Cell Segmentation as Strategic Decision Making
Source: Research (Wash D C). 2026 Jun 1;9:1304. doi: 10.34133/research.1304 (PMC13223358; doi:10.34133/research.1304)
Supplement: Supplementary 1 — Figs. S1 to S10 Table S1 [file research.1304.f1.zip › Supplementary Materials.docx]

**Supp Fig. 1 Evaluation of cell morphologies across different approaches and the noise sampling step of RedeFISH. a.** Comparison of cell area, roundness, and girth distributions among ground-truth (GT) and segmented cells. **b.** Intersection over Union (IoU) of the areas under the density curves of morphological features in **a** between cells segmented by different methods and GT cells. **c.** Spatial distribution of valid and noise transcripts in the Human Lung Cancer (Xenium) dataset, with four regions highlighted for detailed visualization. **d.** Transcript assignment ratio across different regions. **e.** Effect of the noise sampling step on RedeFISH segmentation performance.

**Supp Fig. 2 Comparison of multiple feature dimensions between RedeFISH and alternative approaches. a.** Number of segmented cells for RedeFISH and alternative approaches. **b.** Transcript assignment rates for RedeFISH and alternative approaches. **c.** Percentage difference in transcript assignment ratios for specific genes, relative to the overall ratio for each method on HumanNSCLC (CosMx) and Human Breast Cancer (Xenium) datasets. **d.** Runtime efficiency of RedeFISH, Baysor and Proseg. **e.** Performance of RedeFISH applied to a sequencing-based spatial transcriptomics platform on the Mouse Brain (Stereo-seq) dataset.

**Supp Fig. 3 Spatial distributions of all cell types on human breast cancer (Xenium) dataset through label transferring.** Spatial distributions of 10 cell types by RedeFISH through label transferring. Cyan dots indicate cells belonging to a specific cell type, while gray dots represent all other cells. DCIS, ductal carcinoma in situ; Unk, Unknow.

**Supp Fig. 4 Spatial distributions of all cell types on human-NSCLC (CosMx) dataset through label transferring.** Spatial distributions of 16 cell types by RedeFISH through label transferring. Cyan dots indicate cells belonging to a specific cell type, while gray dots represent all other cells. DCIS, ductal carcinoma in situ. pDC, plasmacytoid dendritic cell; NK, natural killer.

**Supp Fig. 5 Spatial distributions of all cell types on Mouse Ileum (MERFISH) dataset through label transferring.** **a.** Cell-type distribution of all identified cells from RedeFISH, Baysor and staining-based method. **b.** Distribution of indicated cell types based on RedeFISH results. Cyan dots indicate cells belonging to a specific cell type, while gray dots represent all other cells. ICC, Interstitial cells of Cajal.

**Supp Fig. 6 Spatial distributions of all cell types on Mouse Brain (MERSCOPE) dataset through label transferring.** **a.** Cell-type distribution of all identified cells from RedeFISH, Baysor, Proseg, Cellpose-SAM, Stardist and staining-based method. **b.** Distribution of indicated cell types based on RedeFISH results. Cyan dots indicate cells belonging to a specific cell type, while gray dots represent all other cells. Astro, astrocyte; Endo, endothelial cell; Ext, excitatory neurons; Inh, thalamic habenular neuron; LowQ, low quality cells; Micro, microglia; Oligo, oligodendrocyte; OPC, oligodendrocyte precursor cell; Unk, Unknow.

**Supp Fig. 7 Spatial distributions of all cell types on Mouse Liver (MERSCOPE) dataset through label transferring.** **a.** Cell-type distribution of all identified cells from RedeFISH and staining-based method. **b.** Distribution of indicated cell types based on RedeFISH results. Cyan dots indicate cells belonging to a specific cell type, while gray dots represent all other cells. NK, natural killer.

**Supp Fig. 8 Spatial distributions of all cell types on Mouse Brain (Stereo-seq) dataset through label transferring.** **a.** Cell-type distribution of all identified cells from RedeFISH and staining-based method. **b.** Distribution of indicated cell types based on RedeFISH results. Cyan dots indicate cells belonging to a specific cell type, while gray dots represent all other cells. Astro, astrocyte; CA, cornu ammonis area; DG, dentate gyrus; Endo, endothelial cell; EX, excitatory glutamatergic neuron; IN, GABAergic interneuron; Micro, microglia; Oligo, oligodendrocyte; VLMC, vascular and leptomeningeal cells.

**Supp Fig. 9 Analysis of RedeFISH results from Mouse Ileum (MERFISH) dataset. a.** Expression of marker genes for each cell type. **b.** Expression of *Wnt* and *Fzd-Lrp* complexes between stem cell and various cell types. The size of the dots represents the -log_10_(P-values) of the interaction significance. The color of the dots represents the average log-transformed expression of ligand and receptor.

**Supp Fig. 10 Analysis of RedeFISH results from Human Breast Cancer (Xenium) dataset. a.** Pseudotime of DCIS and invasive tumor cells on the tSNE coordinates. **b.** Line chart showing the normalized expression of four selected genes along pseudotime. **c.** Percentage of cell types found in neighboring areas of DCIS or invasive tumor cells. Top: percentages for 18 cell types. Button: percentages for 14 cell types excluding DCIS #1, DCIS #2, DCIS #3 and invasive tumor. **d.** Line chart depicting the proportions of six major cell types in regions located within 10μm, 20μm, 50μm, and 100μm to DCIS or invasive tumor cells along pseudotime.
