# Supplementary figures and images for: Cell Segmentation as Strategic Decision Making
Source: Research (Wash D C). 2026 Jun 1;9:1304. doi: 10.34133/research.1304 (PMC13223358; doi:10.34133/research.1304)

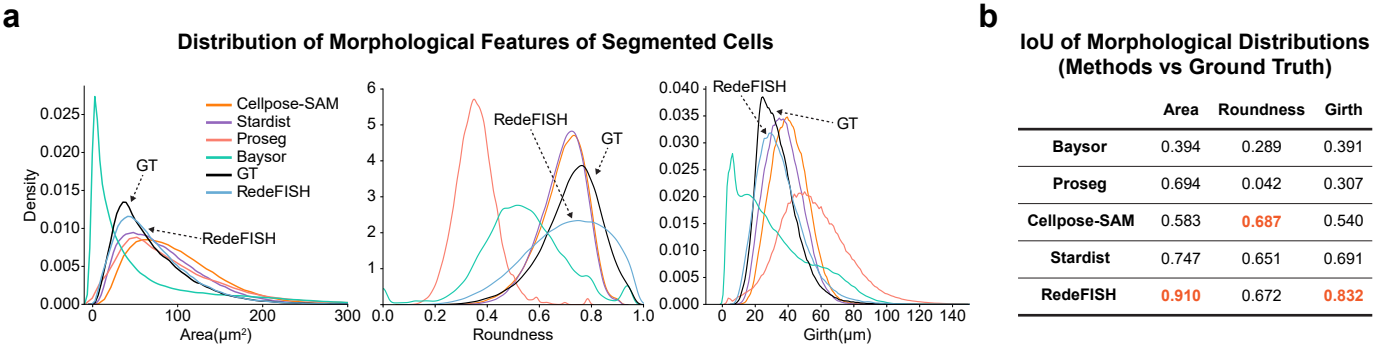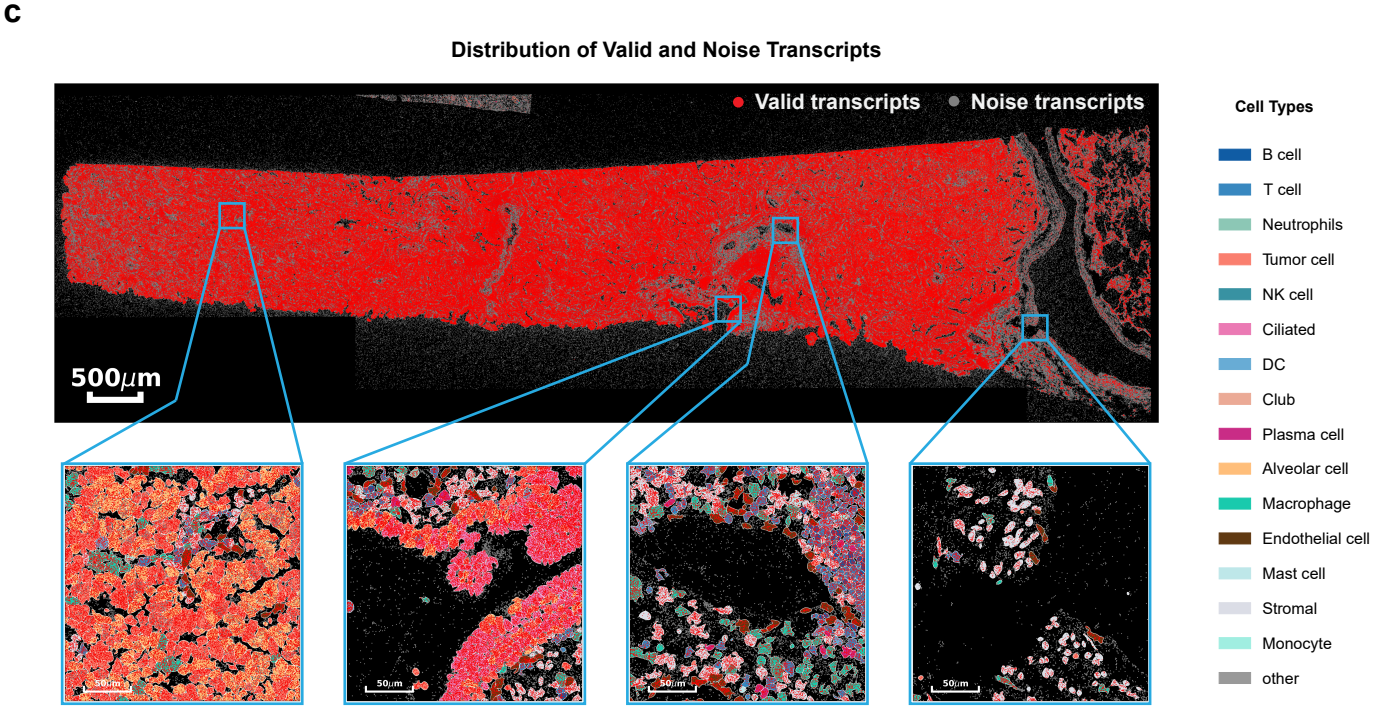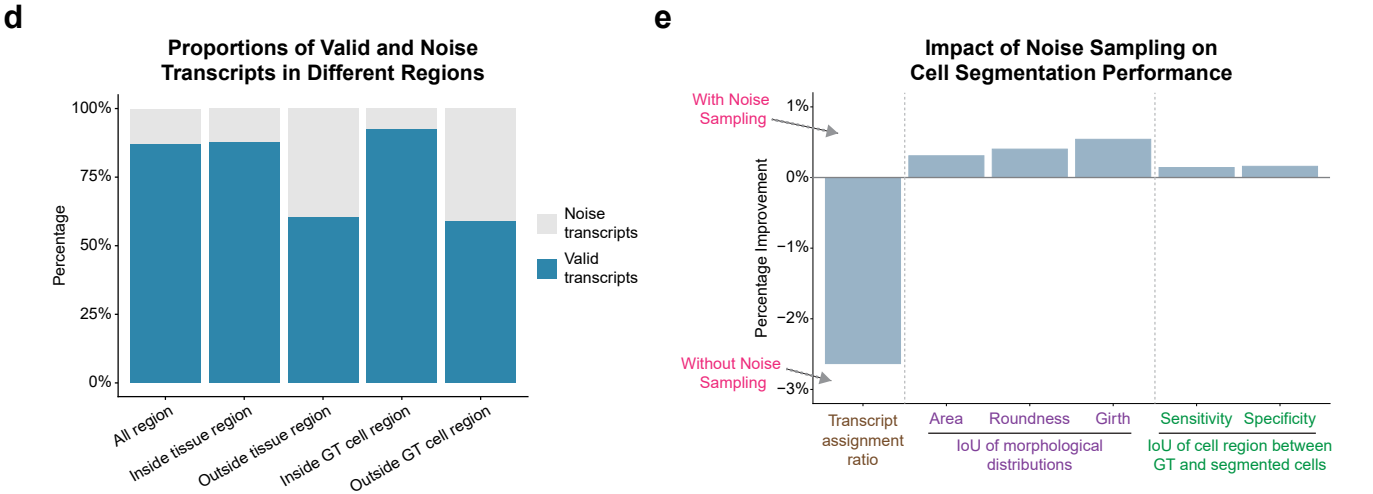

Supplement: Supplementary 1 — Figs. S1 to S10 Table S1 [file research.1304.f1.zip › Supp Fig 1.pdf]

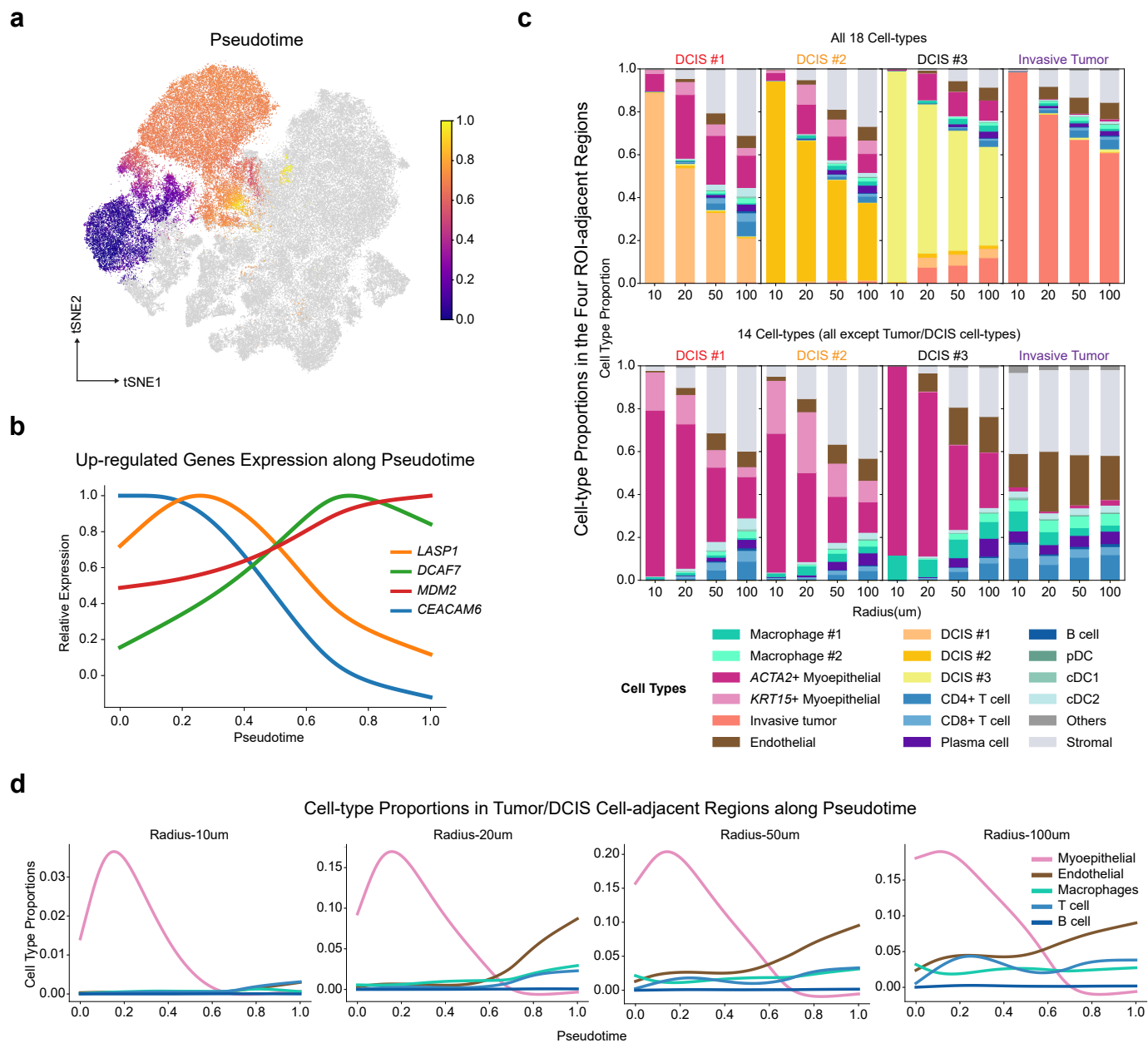

Supplement: Supplementary 1 — Figs. S1 to S10 Table S1 [file research.1304.f1.zip › Supp Fig 10.pdf]

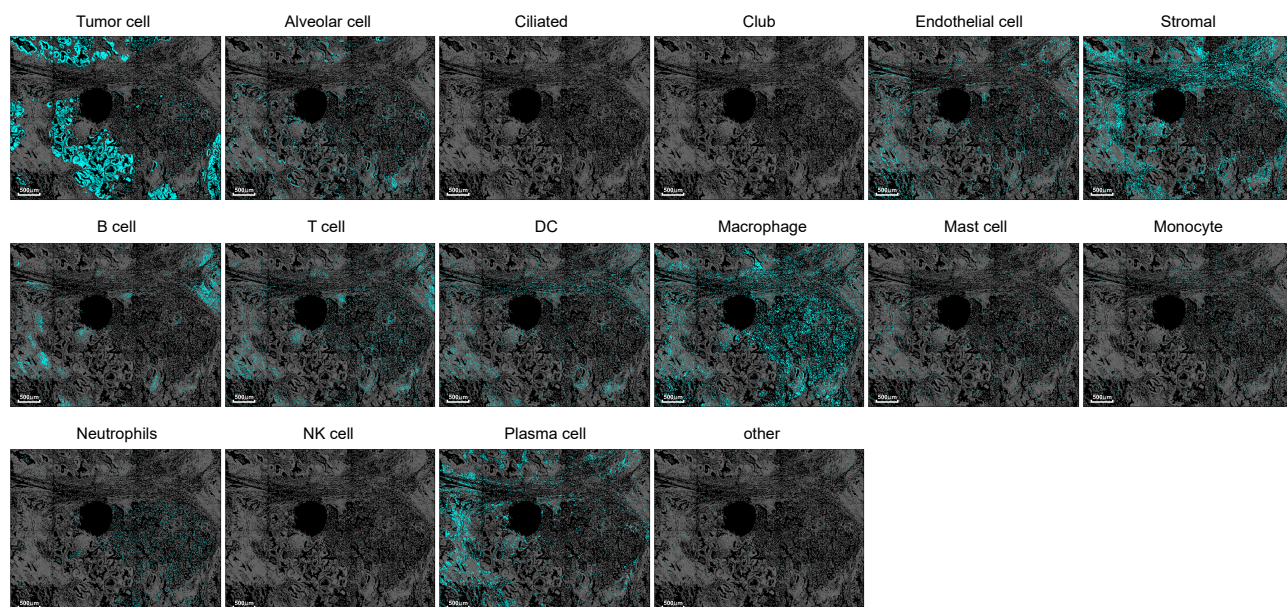

Supplement: Supplementary 1 — Figs. S1 to S10 Table S1 [file research.1304.f1.zip › Supp Fig 4.pdf]

**a**

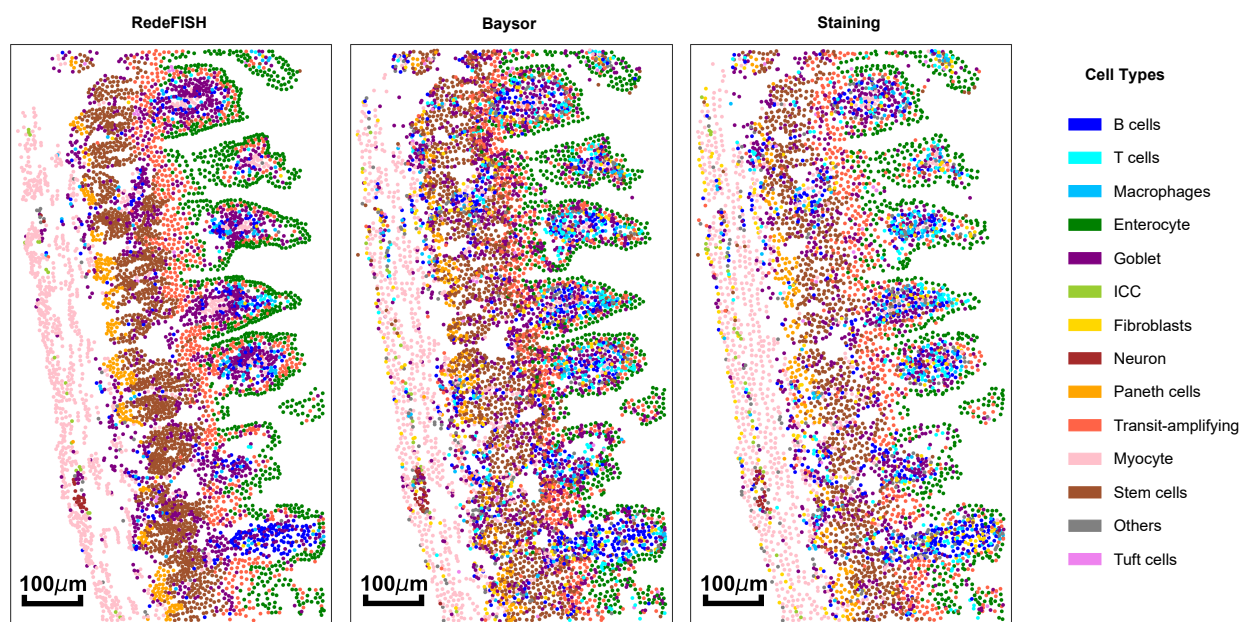

**b**

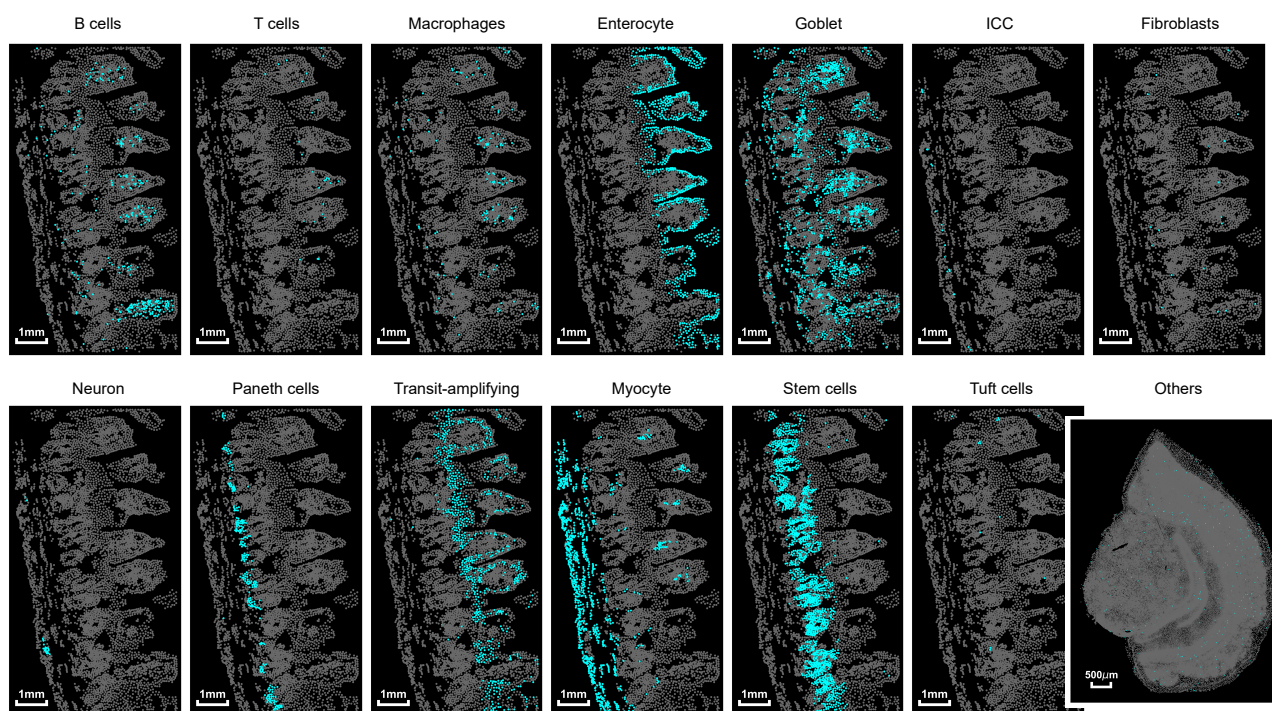

Supplement: Supplementary 1 — Figs. S1 to S10 Table S1 [file research.1304.f1.zip › Supp Fig 5.pdf]

**a**

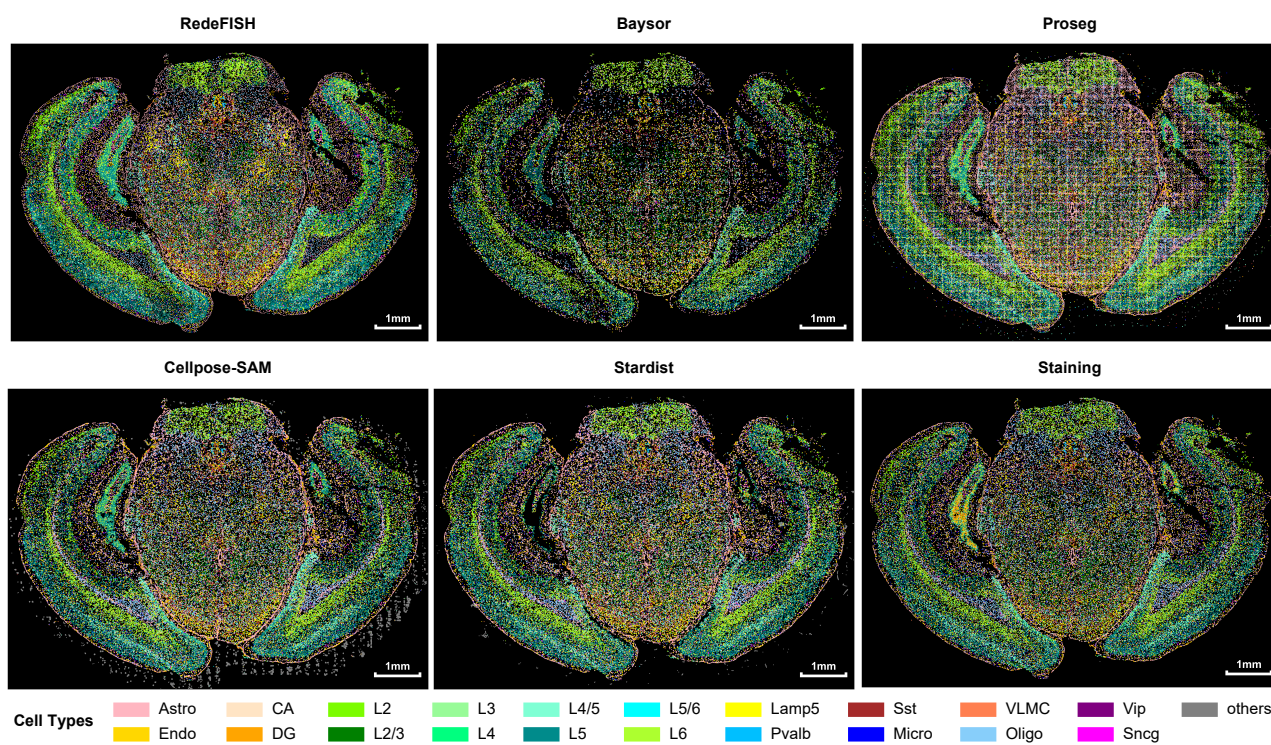

**b**

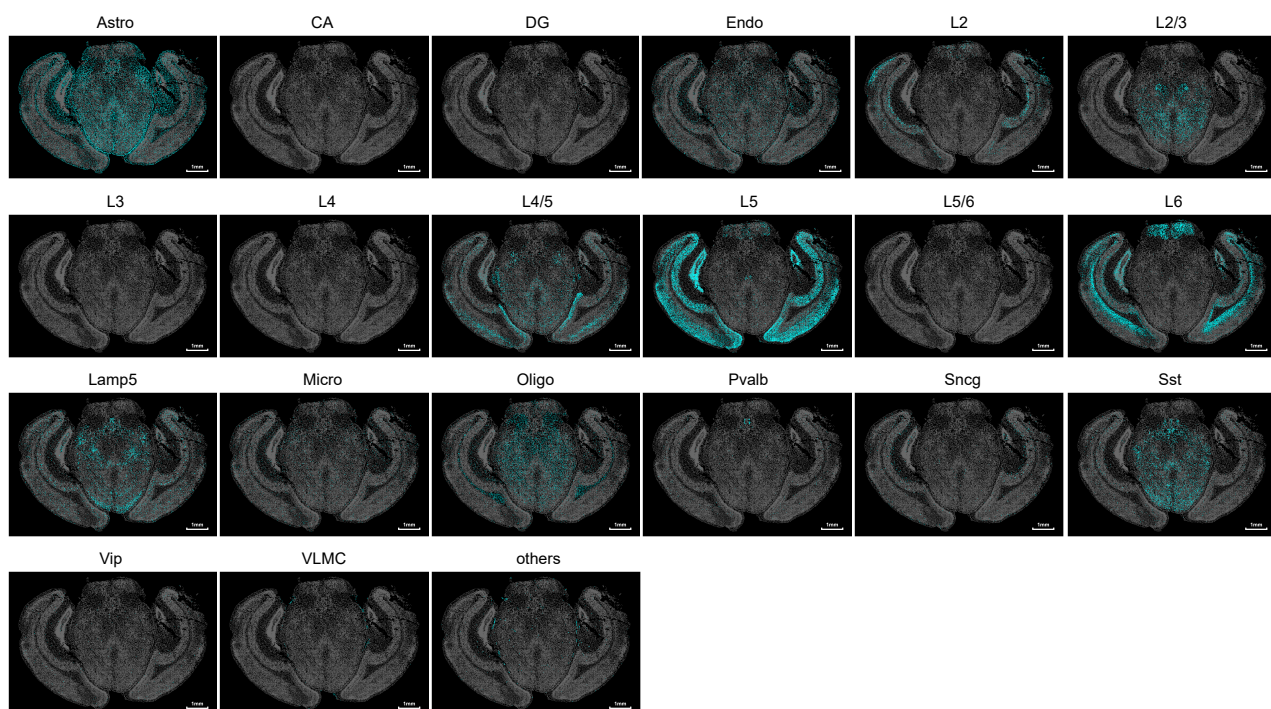

Supplement: Supplementary 1 — Figs. S1 to S10 Table S1 [file research.1304.f1.zip › Supp Fig 6.pdf]

**a**

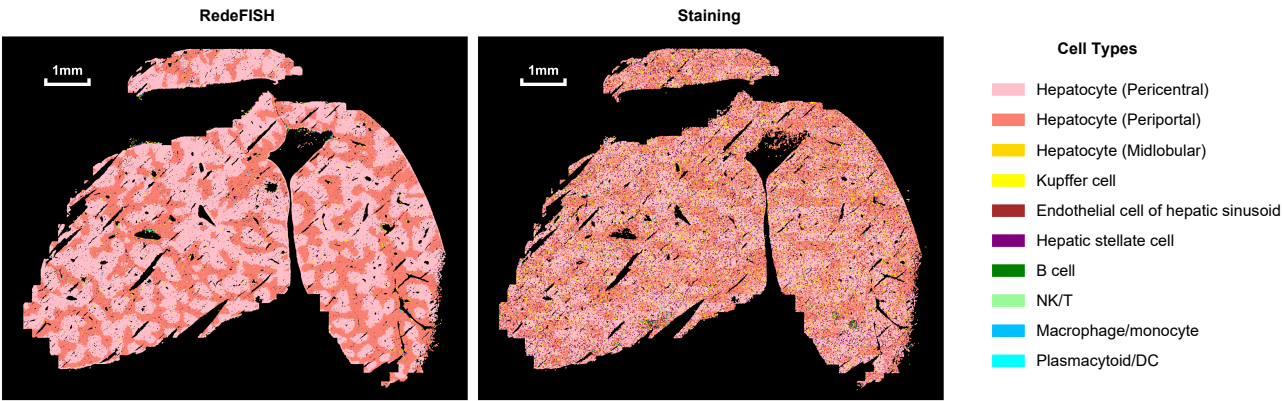

**b**

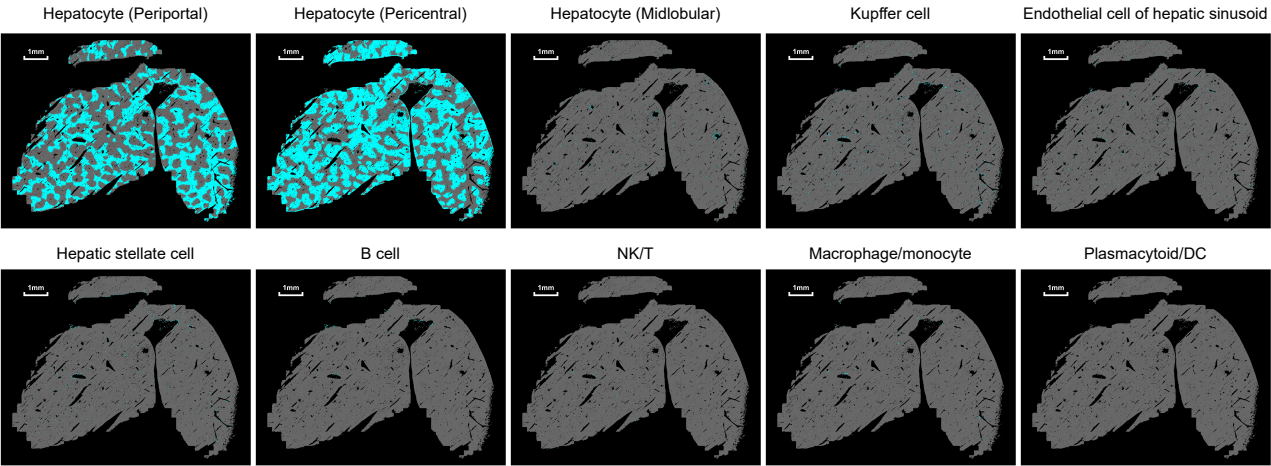

Supplement: Supplementary 1 — Figs. S1 to S10 Table S1 [file research.1304.f1.zip › Supp Fig 7.pdf]

**a**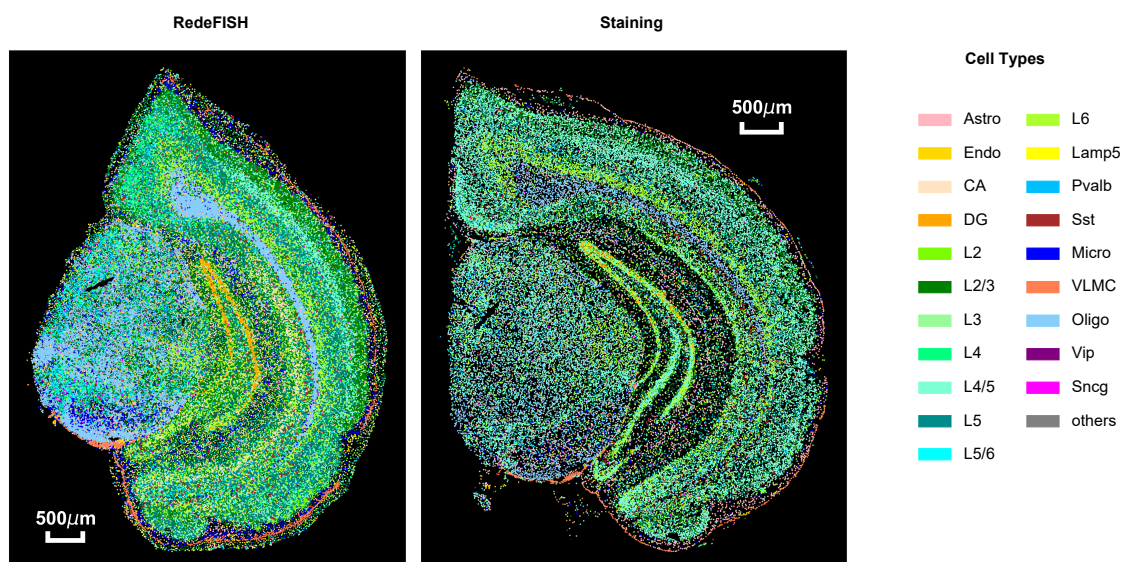**b**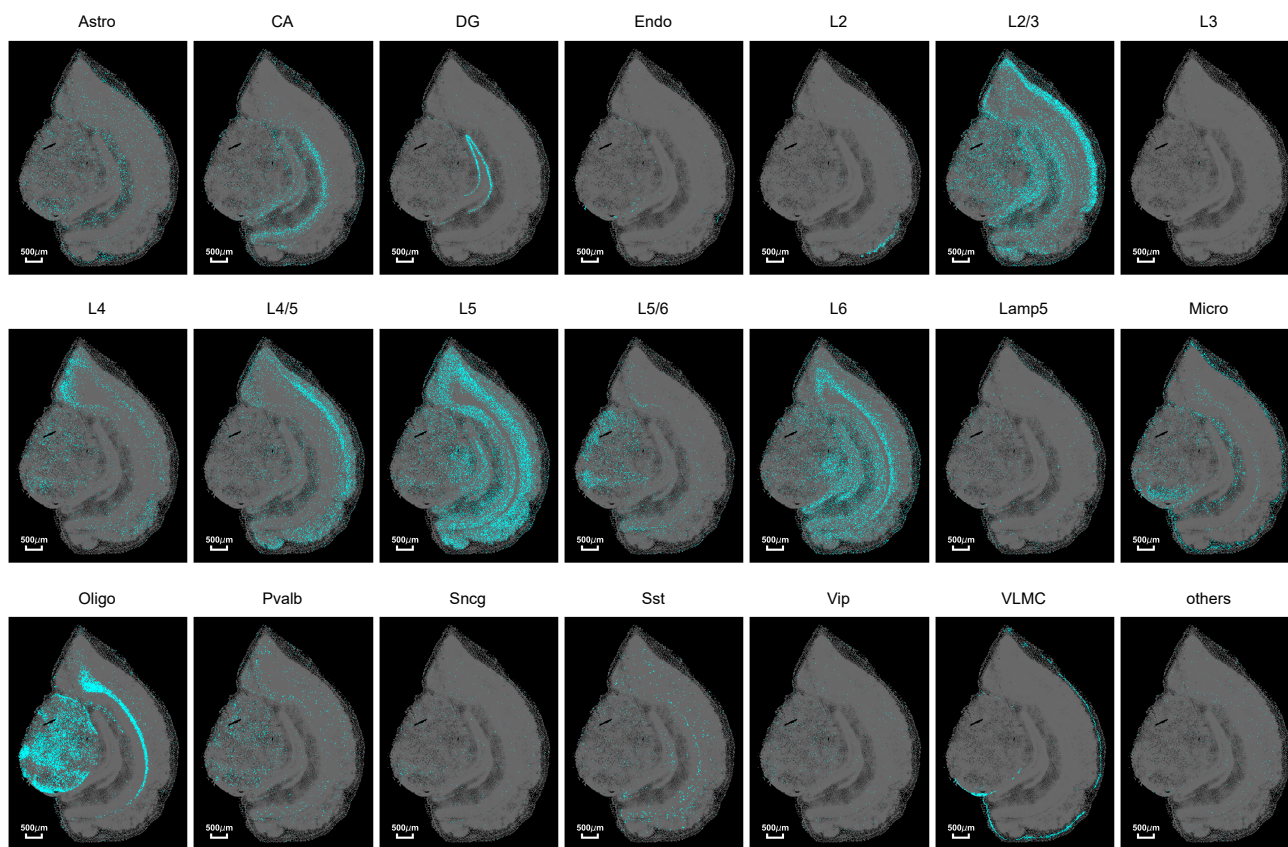

Supplement: Supplementary 1 — Figs. S1 to S10 Table S1 [file research.1304.f1.zip › Supp Fig 8.pdf]

a

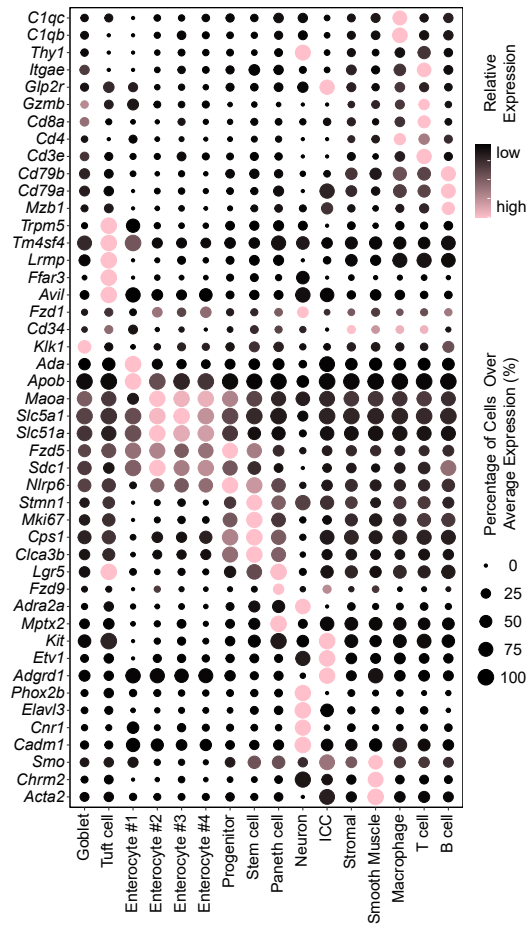

b

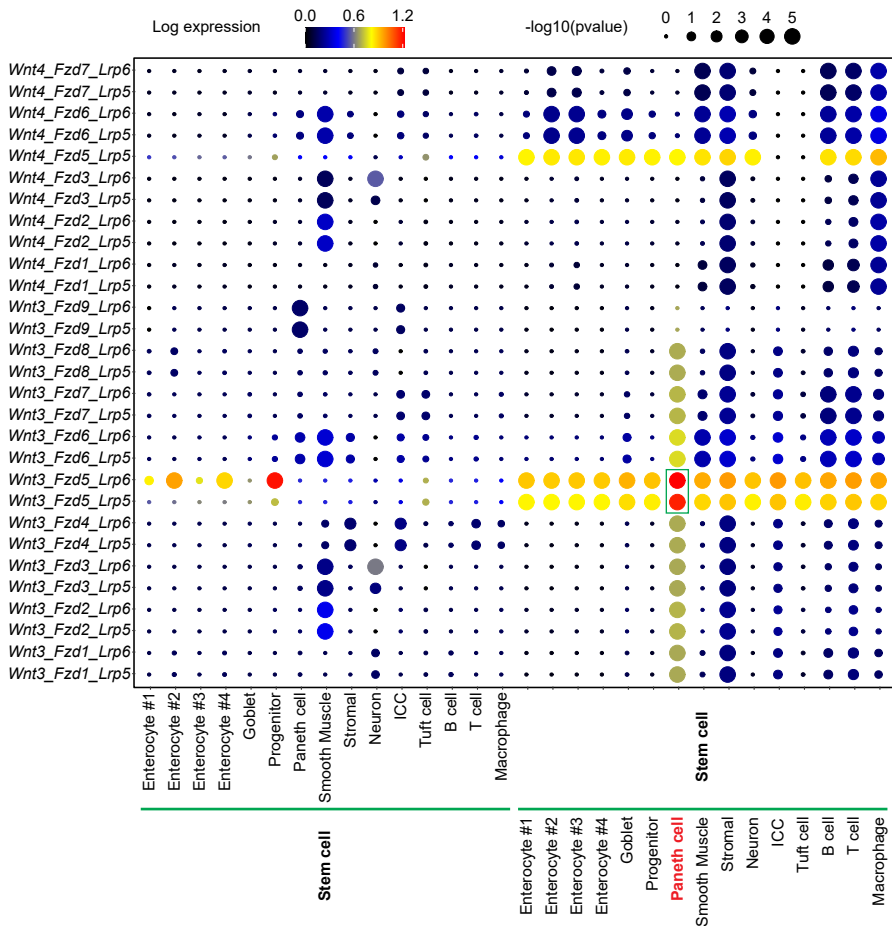

Supplement: Supplementary 1 — Figs. S1 to S10 Table S1 [file research.1304.f1.zip › Supp Fig 9.pdf]
